# Enhanced Protection against Malaria by Indoor Residual Spraying in Addition to Insecticide Treated Nets: Is It Dependent on Transmission Intensity or Net Usage?

**DOI:** 10.1371/journal.pone.0115661

**Published:** 2015-03-26

**Authors:** Philippa A. West, Natacha Protopopoff, Alexandra Wright, Zuhura Kivaju, Robinson Tigererwa, Franklin W. Mosha, William Kisinza, Mark Rowland, Immo Kleinschmidt

**Affiliations:** 1 Department of Infectious Disease Epidemiology, London School of Hygiene and Tropical Medicine, London, United Kingdom; 2 Department of Disease Control, London School of Hygiene and Tropical Medicine, London, United Kingdom; 3 National Institute for Medical Research, Amani Medical Research Centre, Muheza, Tanzania; 4 Department of Health, District Medical Office, Muleba, Tanzania; 5 Kilimanjaro Christian Medical College, Tumaini University, Moshi, Tanzania; 6 MRC Tropical Epidemiology Group, London School of Hygiene and Tropical Medicine, London, United Kingdom; Kenya Medical Research Institute (KEMRI), KENYA

## Abstract

**Background:**

Insecticide treated nets (ITNs) and indoor residual spraying (IRS) are effective vector control tools that protect against malaria. There is conflicting evidence regarding whether using ITNs and IRS in combination provides additional benefit over using either of these methods alone. This study investigated factors that may modify the effect of the combined use of IRS and ITNs compared to using ITNs alone on malaria infection prevalence.

**Methods:**

Secondary analysis was carried out on data from a cluster randomised trial in north-west Tanzania. 50 clusters received ITNs from a universal coverage campaign; of these 25 were randomly allocated to additionally receive two rounds of IRS in 2012. In cross-sectional household surveys children 0.5–14 years old were tested for *Plasmodium falciparum* infections (*Pf*PR) two, six and ten months after the first IRS round.

**Results:**

IRS protected those sleeping under nets (OR = 0.38, 95%CI 0.26–0.57) and those who did not (OR = 0.43, 95%CI 0.29–0.63). The protective effect of IRS was not modified by community level ITN use (ITN use<50%, OR = 0.39, 95%CI 0.26–0.59; ITN use> = 50%, OR = 0.46, 95%CI 0.28–0.74). The additional protection from IRS was similar in low (<10% *Pf*PR, OR = 0.38, 95%CI 0.19–0.75) and high transmission areas (≥10% *Pf*PR, OR = 0.34, 95%CI 0.18–0.67). ITN use was protective at the individual-level regardless of whether the village had been sprayed (OR = 0.83, 95%CI 0.70–0.98). Living in a sprayed village was protective regardless of whether the individual slept under an ITN last night (OR = 0.41, 95%CI 0.29–0.58).

**Interpretation:**

Implementing IRS in addition to ITNs was beneficial for individuals from villages with a wide range of transmission intensities and net utilisation levels. Net users received additional protection from IRS. ITNs were providing some individual protection, even in this area with high levels of pyrethroid insecticide resistance. These results demonstrate that there is a supplementary benefit of IRS even when ITNs are effective.

**Trial Registration:**

ClinicalTrials.gov NCT01697852

## Background

The massive reductions in the burden of malaria over the past decade have been attributed to the scaling-up of insecticide treated nets (ITN), indoor residual spraying (IRS) and effective case management [1]. However, in 2010 there remained an estimated 174 million cases and 596,000 deaths due to malaria in Africa [1]. Universal coverage of the population at risk with long lasting insecticidal nets (LLIN) is recommended by the World Health Organisation (WHO) and most malaria endemic countries in Africa (32/44) have adopted this policy [1,2]. Scaling up of indoor residual spraying (IRS) has been endorsed by WHO since 2006 in Africa [3]. The proportion of at risk populations protected by IRS in Africa has increased from less than 5% in 2005 to 11% in 2011 [1].

In 2011, 30 (of 44) countries/areas in Africa with ongoing malaria transmission used both IRS and ITN in at least some areas to further reduce the malaria burden [1]. Some studies have shown that it is beneficial to use IRS and ITNs in combination compared to using one method alone, [4,5,6,7] but the evidence is inconsistent as other studies have shown no additional benefit [8,9,10]. Factors which might explain the differences in effect include: insecticide used for IRS, IRS coverage, duration between spray rounds, community ITN usage, background malaria transmission, seasonality, insecticide resistance and, confounding with other interventions such as improved diagnosis and treatment or intermittent preventative therapy [4,11,12,13]. To inform national and local planning on the advisability of using LLINs and IRS in combination, it is important to determine what factors may influence the effectiveness and hence cost-benefit of this approach.

Implementing both IRS and universal coverage of ITNs in the same geographical area could reduce malaria transmission by: 1) increasing the proportion of individuals protected by at least one intervention and 2) providing enhanced protection for individuals who are covered by both interventions compared to individuals protected by one method alone [6,13]. How the combination exerts its effect on transmission could be investigated by determining if the addition of IRS is protective for net users or if the benefit of IRS is restricted to those not using ITNs. Observational studies in Equatorial Guinea, Mozambique and Kenya have demonstrated that individuals using ITNs and whose houses have been sprayed received greater protection than those with either method alone [5,6]. Reducing the proportion of those unprotected by either method was shown to be important in a mathematical model which showed that the benefit of a second intervention was greater if it is targeted at those that did not receive the first [12]. If, as this suggests, the most important effect of using the combination of ITNs and IRS is reducing the percentage of the population that is unprotected, it could be argued that, it would be more cost-effective to increase the coverage of one intervention rather than adding a second. Recent WHO policy guidance recommends that a second vector control intervention should not be added to an existing one merely to compensate for inadequate coverage of the first [14]. However reaching high coverage with one intervention may not always be possible.

Tanzania has a high malaria disease burden with over ten million suspected cases reported in 2011 [1]. Malaria control activities have been scaled-up nationally since 2005 [15,16]. A national universal coverage campaign (UCC) distributed LLINs free of charge in 2011 to top-up coverage from previous distributions [15,16]. In 2007 the President’s Malaria Initiative (PMI) funded an IRS programme in Kagera region, north-west Tanzania [17].

This paper investigated: 1) whether ITN coverage, malaria transmission intensity or IRS coverage modify the effect of the combination of IRS and ITNs on malaria infection prevalence; 2) whether IRS additionally benefited those using an ITN, or whether it protected only those who were not using a net; 3) whether IRS and ITNS were both independently effective; and 4) whether the effect of IRS was due to protection at the household or the community level. This was a secondary analysis of data from a cluster randomised trial (CRT) in north-west Tanzania that showed that there was strong additional protection provided by IRS in an area that had benefited from a recent UCC and reached moderate ITN usage (primary analysis of the CRT [4]).

## Methods

The protocol for this trial and supporting CONSORT checklist are available as supporting information; see S1 Study Protocol, S2 Study Protocol, and S1 CONSORT Checklist.

### Study setting

The study area has been described in detail elsewhere [4,18,19]. In summary, it includes 68,346 households from 109 rural villages in Muleba district (1° 45’ S 31° 40’ E), Kagera region, Tanzania, at an altitude ranging from 1100 to 1600m above sea level [18,19]. The two rainy seasons are the “short rains” in October-December with an average monthly rainfall of 160mm and the “long rains” in March-May with an average monthly rainfall of 300mm [20]. Malaria transmission occurs throughout the year peaking after the rainy seasons [21]. *Anopheles gambiae s*.*s*. and *An*. *arabiensis* are the predominant malaria vectors in the area [22]. Annual rounds of IRS with the pyrethroid lambdacyhalothrin (ICON 10CS, Syngenta, Basel, Switzerland) were conducted by the Research Triangle Institute (RTI) in Muleba district to control malaria between 2007 and 2011. Resistance to pyrethroids and DDT, and emerging resistance to carbamates have been reported [22].

### Study design

This study was a secondary analysis of three post-intervention cross-sectional household surveys that were conducted as part of a two-arm CRT in 2012. The trial compared the *Plasmodium falciparum* prevalence rate (*Pf*PR) in children 0.5–14 years old between communities receiving both high coverage IRS and high coverage of ITNs (intervention arm), and communities receiving high coverage of ITNs only (standard-care control arm). A detailed description of the CRT study design has been presented elsewhere [4,18].

The study area was divided into 50 clusters with at least one village per cluster. All clusters received LLINs (Olyset, Sumitomo Chemicals, Japan) from the UCC in 2011 [23,24]. After the UCC 91% of households owned at least one ITN and 58% of households owned enough ITNs to cover all their sleeping places [19]. Twenty-five clusters (50%) were randomly allocated to receive IRS in 2012 in addition to ITNs (IRS+ITN arm). Two rounds of IRS with bendiocarb (400mg/m^2^ of FICAM 80% wettable powder, Bayer Leverkusen, Germany) were conducted between December 2011 and January 2012 (round 1), and between April and May 2012 (round 2).

### Surveys

**Fig 1:**
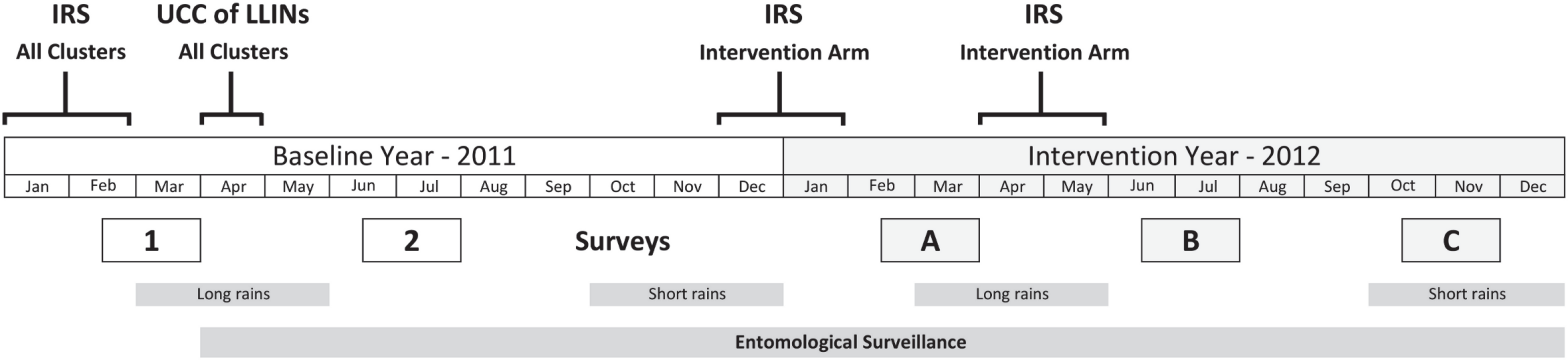
Study timetable. Note: Surveys 1 and 2 are baseline surveys. Surveys A, B and C are post intervention.

The study timetable (Fig. 1) shows the timing of the surveys, the rainy seasons and the spray rounds. Survey A (23^rd^ February to 31^st^ March) was undertaken after the short rainy season, two months after spray round one. Survey B (25^th^ June—31^st^ July) was after the long rainy season, six months after spray round one and two months after spray round two. Survey C (25^th^ October—4^th^ December) was at the beginning of the short rains after the long dry season, six months after spray round two and ten months after spray round one [4]. Background malaria transmission rates for each cluster were estimated by baseline surveys conducted in 2011 during the same periods as surveys A and B.

For each survey 4,000 households were randomly selected (80 per cluster). Households were eligible for inclusion if they had residents aged 6 months to 14 years old. After seeking written informed consent, the household head or another resident adult was interviewed. An adapted version of the standard Malaria Indicator Survey [25] was used to gather data on household demographics, bed net ownership and usage, IRS coverage, and other household characteristics including socio-economic indicators.

Children aged 0.5–14 years old were eligible for the study. Up to three children per household were randomly selected to be tested for malaria parasites using rapid diagnostic tests (RDT) (CareStart (Pan) Malaria DiaSys, Wokingham, UK). This selection process was estimated to result in approximately 4,000 children being tested per survey (80 per cluster). Sample size was calculated for the main trial objectives [4]. Individuals testing positive by RDT were treated with artemether/lumefantrine (Artefan 20/120, Ajanta Pharma Ltd, Maharashtra, India) following national treatment guidelines [26].

### Statistical analysis

All analysis was carried out in Stata 12 (Statacorp, Texas, USA). The outcome for all analyses was *Pf*PR in children 0.5–14 years old. *Pf*PR was considered as *P*. *falciparum* alone or in mixed infections as detected by the RDT. All statistical inference allowed for within cluster correlation of responses by using a robust variance estimator to calculate standard errors (Stata survey commands, first-order Taylor-series linearization method) [27,28]. For each cluster the mean *Pf*PR from the two baseline surveys was used as a proxy for baseline malaria transmission intensity to control for differences between clusters. This was used as a continuous variable in the final model but was categorised into low transmission, *Pf*PR <10%, and high transmission, ≥10%, to investigate interactions. A socio-economic status (SES) score for each household was determined using principal component analysis of the following 11 household-level variables: number of rooms, household crowding, level of schooling of the household head, house construction (including floor, wall, and roof materials) and ownership of livestock, farmland, bicycles, mobile phones and radios [29,30]. Household SES quintiles were generated from the scores.

Two multi-variable logistic regression models were used in the analysis. Model 1 aimed to determine the effect of spraying at the village-level and the individual effect of using an ITN, adjusted for each other and for confounders. Village-level IRS status (sprayed/not sprayed) and individual use of an ITN the previous night to the survey were included in this model as the main risk factors of interest. For this analysis all surveys were combined and the survey identifier was included to account for the differences between the surveys (proxy for season and time since IRS). Associations between *Pf*PR and the following factors were investigated to determine if they would be included in the model as co-variates: household SES, individual age and baseline malaria transmission. The three factors were retained in the model because they were associated with *Pf*PR in uni-variable analysis (p<0.1) and after adjusting for the other factors in the model (p<0.05).

Associations between *Pf*PR and the following were investigated and were retained in the model if p<0.05: community net usage (% of residents using an ITN last night, categorised as <50% and ≥50%), community universal coverage of nets (% of households with ≥1 ITN per 2 residents) and household universal coverage of nets (households with ≥1 ITN per 2 residents). Possible interactions were explored between village (community) spray status and the following: community baseline malaria transmission intensity, individual net use and community net use.

Model 2 was based on the first model and aimed to investigate:

1. Whether the effect of IRS on *Pf*PR in addition to ITNs, varied with community spray coverage
2. Whether the effect of IRS on *Pf*PR was due to:
  - Individuals being directly protected by their house being sprayed (household-level protection)
  - Individuals being protected by living in a community with high spray coverage (community-level protection) or
  - A combination of the two
3. Whether the protectiveness of individual net use was the same in high and low spray coverage areas.

The second model excluded the variable for sprayed village but instead included community spray coverage and household IRS status (reported sprayed in last round versus not sprayed in the last round). Community spray coverage was categorised as follows: village not sprayed i.e. control arm in the original CRT; sprayed village with <90% of households reported sprayed in the last spray round; and sprayed villages with ≥90% of households reported sprayed in the last spray round. Spray coverage and village spray status could not be included in the same model as they were closely correlated.

Complete record analysis only was undertaken, i.e. there was no imputation for missing data.

### Ethics Statement

The trial was approved by the ethics review committees of the Kilimanjaro Christian Medical College, the National Institute for Medical Research Tanzania and the London School of Hygiene and Tropical Medicine (approved August-September 2010). Written informed consent was obtained from a guardian for each child enrolled in the study. The trial is registered with ClinicalTrials.gov (registration number NCT01697852) in September 2012. The trial was not registered earlier because the authors were not aware of journal requirements for prospective registration. The authors confirm that all ongoing and related trials for this drug or intervention are registered.

## Results

### Descriptive information

**Fig 2:**
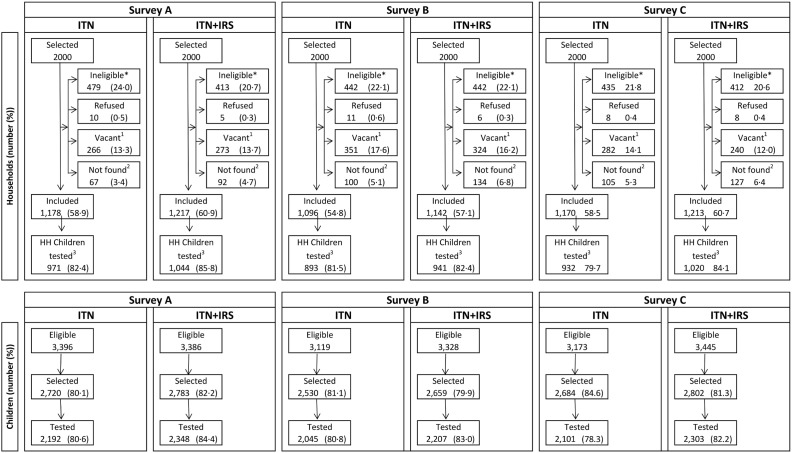
Trial profile for study households and children in the ITN only and ITN+IRS study arms. Note: Survey A = 2 months after first intervention spray. Survey B = 6 months after first intervention spray and 2 months after second intervention spray. Survey C = 10 months after first intervention spray and 6 months after second intervention spray. *No children 0.5–14 y old. ^1^Dwelling vacant for survey duration. ^2^Includes not found (91.0%), not visited (2.4%), and missing data (6.6%). ^3^Households (HH) that were included and where children attended for testing.

A detailed flow diagram showing study participation was presented in the main trial paper [4] and is shown in Fig. 2. In summary, 22% of households selected had no children between 0.5 and 14 years old (were ineligible for study participation), 15% were vacant on the day of survey, 0.4% refused and 59% agreed to participate in the survey [4]. Of the children sampled, 13,198 (82%) were tested, of which 13,146 (99.6%) had a valid RDT result. 12,933 and 12,913 individuals had the complete data required for model 1 and 2 respectively.

IRS coverage reported by householders in the sprayed villages was 92·1% after the first spray round and 89·5% after the second. Overall 84·6% of households owned at least one ITN. ITN usage in children was similar between sprayed and unsprayed villages but declined from 50% in survey A to 36% in survey C. More details are given elsewhere [4,18,19]. Of all children in the study, 29.1% (95% CI 22.1–37.4) were not directly protected by either sleeping under an ITN the previous night or living in a household sprayed in the last round. This reduced to 5.8% (95% CI 4.1–8.0) not directly protected when considering only the sprayed villages. Overall 22.5% of children (95% CI 17.1–29.1) were directly protected by both ITN use and living in a sprayed household. This increased to 40.5% (95% CI 36.4–44.7) when considering only the sprayed villages.

### Effectiveness of additional IRS

The odds ratio (OR) for *Pf*PR in sprayed versus unsprayed villages (as randomised), adjusted for survey was 0.43 (95% CI 0.19–0.97; p = 0.04) as previously reported [4]. This OR was very similar (OR 0.41) after adjusting for individual net use, household SES, baseline malaria prevalence, age, household SES and survey (Table 1), but the strength of evidence for the association increased (Model 1: p<0.0001) and hence the 95% CI was reduced (0.29–0.58). The effect of being in a sprayed village did not vary significantly between high transmission (baseline prevalence ≥10%, OR 0.34, 95% CI 0.18–0.67) and low transmission areas (<10%, OR 0.38, 95% CI 0.19–0.75) (results not tabulated: p-value for interaction = 0.84; adjusted for individual net use, survey, SES and age). There was still no evidence for interaction when high transmission areas were categorised as being ≥20% baseline prevalence (p = 0.97).

**Table 1:** Model 1: Effect of village IRS and individual net use on *Pf*PR, adjusted for other risk factors, Muleba 2012.

	*PfPR^1^*	Unadjusted Odds Ratio^2^	Adjusted Odds Ratio^3^
	%, [95% CI], (n)	OR, [95% CI], p-value	OR, [95% CI], p-value
**Study arm**			
*ITN only*	26.1, [16.7,38.4], (6315)	1.00	1.00
*ITN+IRS*	13.3, [7.9,21.5], (6831)	0.43, [0.19–0.97], p = 0.0434	0.41, [0.29–0.58], p<0.0001
**Individual net use^4^**			
* No*	19.1, [13.4–26.5], (7511)	1.00	1.00
*Yes*	19.9, [13.6–28.1], (5635)	1.05, [0.84–1.32], p = 0.0428	0.83, [0.70–0.98], p = 0.0305
**Survey^5^**			
*Post Intervention A*	18.4, [13.3–25.0], (4533)	1.00	1.00
*Post Intervention B*	21.3, [14.9–29.4], (4237)	1.20, [0.99–1.45]	1.23, [0.92–1.65]
*Post Intervention C*	18.7, [12.3–27.4], (4376)	1.02, [0.83–1.26], p = 0.0551	0.87, [0.66–1.15], p = 0.0525
**Baseline malaria prev^6^**			
*per 10% increase*		2.05, [1.85–2.28], p = <0.0001	2.04, [1.85–2.25], p = <0.0001
**Individual age (years)**			
*0.5–4*	18.1, [12.4–25.6], (4745)	1.00	1.00
*5–9*	21.1, [14.8–29.2], (4819)	1.21, [1.06–1.39]	1.56, [1.32–1.83]
*10–14*	19.0, [13.3–26.4], (3582)	1.07, [0.88–1.29], p<0.0001	1.57, [1.28–1.91], p<0.0001
**Household SES (Quintiles)**			
*1—poorest*	31.6, [24.0–40.3], (2358)	1.00	1.00
*2*	23.9, [16.8–32.8], (2696)	0.68, [0.58–0.79]	0.79, [0.68–0.92]
*3*	19.9, [13.6–28.0], (2675)	0.54, [0.45–0.63]	0.68, [0.55–0.83]
*4*	15.3, [10.4–21.8], (2658)	0.39, [0.31–0.50]	0.67, [0.52–0.86]
*5—least poor*	7.1, [4.8–10.5], (2546)	0.17, [0.11–0.24], p<0.0001	0.43, [0.34–0.54], p<0.0001

Note: ^1^ *Pf*PR = *Plasmodium falciparum* infection prevalence. ^2^ Only adjusted for survey. ^3^Adjusted for all other factors in the table. ^4^Reported sleeping under an ITN the previous night. ^5^ CI = Confidence interval. ^6^Mean cluster *Plasmodium falciparum* infection prevalence from the two baseline surveys in 2011. N = Number tested. Survey A = 2 months after 1st intervention spray. Survey B = 6 months after 1^st^ intervention spray and 2 months after 2^nd^ spray. Survey C = 10 months after 1^st^ intervention spray and 6 months after 2^nd^ spray.

The reduction in *Pf*PR associated with living in a sprayed village was similar among ITN users (OR 0.38, 95% CI 0.26–0.57) and non-users (OR 0.43, 95% CI 0.29–0.63) after adjusting for survey, baseline malaria prevalence, SES and age (results not tabulated: p-value for interaction = 0.48). There was no evidence to suggest that the reduction in *Pf*PR associated with being in a sprayed village was different between communities where ≥50% (median = 55%) of residents used ITNs (OR 0.46, 95% CI 0.28–0.74) and communities where <50% (median = 37%) of residents used ITNs (OR 0.39, 95% CI 0.26–0.59, p-value for interaction = 0.6). Increasing the cut-off for high community net usage from 50% to 60% did not change this result.

### Effectiveness of ITNs

Individual ITN use was associated with a lower *Pf*PR (OR 0.83, 95% CI 0.70–0.98; p = 0.03) after adjusting for village IRS status, background malaria prevalence and other confounders (Table 1, Model 1). There was no evidence to suggest that the protection gained from using a net differed between unsprayed (OR 0.87, 95% CI 0.68–1.12) and sprayed villages (OR 0.78, 95% CI 0.63–0.97, Model 1: p-value for interaction = 0.48, adjusted for SES, age, survey, background malaria). The effect of ITNs was similar in villages with spray coverage <90% (OR 0.84, 95% CI 0.63–1.10) and villages with spray coverage ≥90% (OR 0.74, 95%CI 0.56–0.97, p-value for interaction = 0.59). Community net usage was not associated with *Pf*PR after adjusting for individual net use and other factors (Model 1: results not tabulated, p = 0.38). Household net ownership and community net ownership were investigated as a proxy measure for net use because there can be problems with mis-reporting of net use [18,31]. Neither variable was associated with *Pf*PR in uni-variable analysis or after adjusting for other factors (all p-values > 0.2).

### Individuals with ITNs and IRS

The odds of infection for individuals who live in a sprayed village and who use ITNs is two thirds lower than those with neither intervention (calculated from model 1, OR 0.34, 95% CI 0.23–0.53, p = <0.001). This is a greater reduction than using nets alone (OR 0.83) but is only slightly greater than to the reduction associated with living in an IRS village (OR 0.41) (Table 1).

### Community and household effect of IRS

Model 2 showed that community spray coverage and household spray status were both independently associated with *Pf*PR after adjusting for other co-variates (Table 2). Living in a sprayed house was associated with a one third reduction in odds of infection (OR 0.67, 95% CI 0.49–0.91), regardless of spray coverage. Compared to unsprayed areas the odds of infection were a third less in communities with spray coverage less than 90% (OR 0.68, 95% CI 0.44–1.06) and were halved in clusters with at least 90% spray coverage (OR 0.50, 95% CI 0.31–0.82), regardless of whether the house was sprayed or not. Median coverage was 81% in communities with coverage <90% and was 95% in clusters with coverage ≥90%. There was, however, only weak evidence that individuals in communities with IRS coverage ≥90% (median = 91%) were better protected than those in communities with <90% coverage (interaction OR 0.74, p = 0.069).

**Table 2:** Model 2: Household and community level effect of IRS, and individual net use on *Pf*PR (as per protocol analysis), Muleba 2012.

	*PfPR* ^*1*^	Unadjusted Odds Ratio^2^	Adjusted Odds Ratio^3^
	%, [95% CI], (n)	OR, [95% CI], p-value	OR, [95% CI], p-value
**House sprayed in last round^4^**			
*No*	27.0, [18.0–38.5], (6489)	1.00	1.00
*Yes*	12.0, [7.5–18.8], (6631)	0.37, [0.18–0.74], p = 0.0123	0.67, [0.49–0.91], p = 0.012
**Community Spray coverage**			
*Houses not Sprayed (Control arm)*	26.7, [17.1–39.3], (6104)	1.00	1.00
*<90% of houses Sprayed*	21.8, [10.5–39.8], (2066)	0.76, [0.27–2.17]	0.68, [0.44–1.06]
*> = 90% of houses Sprayed*	9.5, [5.5–16.0], (4976)	0.29, [0.13–0.66], p = 0.026	0.50, [0.31–0.82], p = 0.026
**Individual net use**			
*No*	19.1, [13.4–26.5], (7511)	1.00	1.00
*Yes*	19.9, [13.6–28.1], (5635)	1.05, [0.84–1.32], p = 0.0428	0.84, [0.72–0.99], p = 0.043

Note: ^1^ *Pf*PR = *Plasmodium falciparum* infection prevalence from RDTs. ^2^Unadjusted odds ratios are only adjusted for survey. ^3^Adjusted for all other factors in the table and: household SES, individual age, baseline malaria prevalence and survey. ^4^HH = Household. CI = Confidence interval. N = Number tested.

## Discussion

This study showed that: 1) the protection provided by IRS was not limited to individuals who were unprotected by nets as the effect of IRS was the same in net users and non-users; 2) the protective effect of IRS was not modified by community level ITN use; 3) the additional protection from IRS was similar in low and high transmission areas; 4) ITNs were protective after accounting for the effect of IRS; 5) individuals who reported using both IRS and ITN together were more protected than those using ITNs alone; and 6) IRS provided protection both at the household-level to those living in sprayed houses, and at the community-level to those living in sprayed communities, even if their particular dwelling was not sprayed.

These results demonstrate that the supplementary use of IRS is not enhancing protection by purely reducing the proportion that are otherwise unprotected due to incomplete coverage of ITNs, but further reduces the malaria risk in all individuals, including those using ITNs. The evidence from this study is that ITNs were providing individual protection against malaria, even in this area with high levels of pyrethroid resistance [22]; the supplementary benefit of IRS was therefore not because ITNs were ineffective.

IRS effectiveness did not vary with background malaria transmission within the study area; *Pf*PR in the clusters varied from 1.3% to 60.8% at baseline (mean cluster *Pf*PR from two baseline surveys). This suggests that a benefit would be gained from using IRS in addition to nets in both low and high transmission areas. There is evidence from non-randomised studies that the combination of IRS and ITNs provides greater protection than using only one alone in high transmission areas such as Kenya and Equatorial Guinea [5,6]. Observational studies in Eritrea and Ethiopia, where malaria endemicity is low, showed that both IRS and ITN use were independently effective when used in the same area [31,32].

There was no evidence in the current study that the additional benefit of IRS varied with individual or community ITN usage. However, in this study it could be argued that the “high ITN usage” clusters were actually only “moderate usage” because the median value in the category was 55% and the maximum was 69.5%. Further work is needed to determine if IRS would still enhance protection against malaria if ITN usage was very high.

In this study the consistency of the protective effect of IRS suggests that individual net use, and differences in community net usage or background malaria transmission, of the scale seen within this study, may not be important in determining why the combination has been shown to be more effective than one intervention alone in some studies but not in others [6,8,9,13]. However there are some limitations to the analysis presented here. One obvious limitation is the lack of statistical power to detect interactions because this was not taken into account in sample size calculations for the trial [33]. A second is that intervention coverage levels and malaria transmission levels investigated were limited to those present within the study area. A third is that the effect of confounding cannot be ruled out as the factors investigated were not randomly allocated (e.g. community net usage). These limitations mean that the levels of community net use and malaria transmission intensity cannot be ruled out as possible determinants for whether the combination of IRS and ITNs provides enhanced protection against malaria. There are other factors that may determine the effectiveness of the combination that could not be investigated in this study, these include: insecticide type, spray interval and the primary malaria vector. The effect of IRS in this study would have been optimised by the short interval between spray rounds of only four months. The residual efficacy of bendiocarb is reported as two to six months [34]. The spraying was timed to provide protection during the two annual peaks in malaria transmission but this meant that the interval was shorter than is usual practice. A CRT in Benin, that also compared bendiocarb-IRS and ITNs to ITNs alone, found no difference in malaria incidence between these two groups, but the spray interval was 8 months [9].

Accounting for differences in malaria transmission at baseline, individual net use, age, and household poverty did not change the odds ratio for *Pf*PR in sprayed villages compared to unsprayed villages. This suggests that the two study arms were well balanced in terms of these risk factors for malaria. The precision of the estimate was greater after adjusting for these factors (p-value reduced from 0.04 to <0.0001) due to the heterogeneity between clusters in this study area.

The majority of villages in the study area had high IRS coverage. IRS was effective in villages with coverage of <90% (median coverage 81%) and there was weak evidence that the effect of IRS was greater in villages with at least 90% coverage. Rehman et al [35] found that IRS was not protective when spray coverage was <80% (50 and 79.9%) in Equatorial Guinea. The “low coverage” group in the current study was defined as spray coverage of <90% and the distribution was skewed towards higher coverage with 16 out of the 23 (70%) clusters having spray coverage of at least 80%. Therefore the “low coverage” groups in the two studies are not comparable. The higher cut-off of 90% coverage was required in this study due to the overall high spray coverage and thus the small number of clusters with coverage below 80%.

IRS primarily reduces malaria transmission at the community-level by reducing mosquito longevity and abundance, but it has also been reported to provide household-level protection [36,37,38]. In this study there was evidence that living in a sprayed household (direct-effect) was associated with a reduction in odds of *Pf* infection regardless of community spray coverage. Although bendiocarb has been reported as having low irritancy and excito-repellency, some studies have reported reduced house entry, endophily and endophagy due to bendiocarb-IRS [37,39,40]. An alternative explanation is that the association between household spray status and *Pf*PR was confounded by other factors. Firstly, a sprayed household could be more likely to be surrounded by sprayed households and thus the apparent household-level protection could be a community-level effect of IRS on a smaller scale than the cluster. Secondly, households in remote locations are often poor quality with easy mosquito entry, and these households may be less likely to be sprayed. This second potential confounder was accounted for to some extent by including household SES in the model and there was no evidence to suggest that SES confounded the relationship between *Pf*PR and household IRS.

The effectiveness of ITNs for preventing malaria is well established [41] but the epidemiological impact of insecticide resistance on the protectiveness of ITNs is currently unknown [38]. Despite a high level of pyrethroid resistance in malaria vectors in this area [22] individuals who reported using ITNs were protected from *Pf* infection. The reduction in odds of almost one fifth (OR = 0.83) is similar to the estimated protective efficacy of 13% against parasite prevalence in stable transmission areas in the Cochrane review of ITNs [41]. The effectiveness of ITNs was the same in areas with and without IRS, and was not affected by IRS coverage. Non-randomised studies in Equatorial Guinea, Sao Tome and Principe, Kenya and Mozambique also showed a reduced risk of malaria infection in those protected by both IRS and ITNs compared to IRS alone [5,6,7,42,43].

The protection provided by IRS in this study appeared to be substantially greater than that provided by ITNs (adjusted ORs 0.41 versus 0.83). Although the protective effect of ITNs in this study was similar to the effectiveness calculated in a meta-analysis of studies from stable malaria transmission areas [41], it could have been underestimated or suboptimal for the following reasons. Firstly, compliers were compared to non-compliers which means that the community protection provided by ITNs would not be included in this estimate and that there could be confounding because ITN use was not randomly allocated. Secondly, there were relatively low levels of ITN use in this study, suggesting that the community protection provided by the ITNs would have been suboptimal [44,45,46,47,48]. The high level of pyrethroid resistance may also have compromised the protective effect of the ITNs if the mortality rate due to the insecticide was reduced. There was no evidence for a community-level effect of ITN use when low coverage areas were compared to moderate coverage areas, but the study lacked power for detecting community protection, since nets were distributed in all areas.

### Conclusion

This analysis suggests that the addition of IRS in an area already using ITNs is beneficial in villages with high or moderate malaria transmission, and with low or moderate community net usage. More research is needed to confirm these results at high levels of ITN use, but it is difficult to design trials with the primary objective of investigating whether intervention coverage or malaria transmission intensity modify the effectiveness of the combination of IRS and ITNs. IRS was additionally beneficial to ITN users. This study demonstrated that ITNs are beneficial in this area, even when used in combination with high coverage IRS, and with high levels of pyrethroid resistance. Children receiving both interventions were more protected than those with ITNS alone. Although both IRS and ITNs have been shown to be cost effective, studies are needed to estimate the cost-effectiveness of the combination [2,49,50,51]. Based on current evidence the use of IRS with bendiocarb and combined with ITNs, is beneficial in a wide range of settings, compared to implementing either of these interventions alone.

## Supporting Information

S1 CONSORT Checklist CONSORT Checklist. (DOCX)

S1 Study Protocol Study protocol. (PDF)

S2 Study Protocol Study protocol addendum. (PDF)
