# Supplementary material for: Enhanced Protection against Malaria by Indoor Residual Spraying in Addition to Insecticide Treated Nets: Is It Dependent on Transmission Intensity or Net Usage?
Source: PLoS One. 2015 Mar 26;10(3):e0115661. doi: 10.1371/journal.pone.0115661 (PMC4374910; doi:10.1371/journal.pone.0115661)
Supplement: S1 Study Protocol — (PDF) [file pone.0115661.s002.pdf]

The combined use of indoor residual spraying (IRS) and long-lasting insecticidal nets (LLINs) for malaria reduction in endemic rural Tanzania

Detailed Work Plan (16 March 2011)

Primary Recipient

London School of Hygiene and Tropical Medicine
Keppel Street
London
WC1E 7HT
United Kingdom
Telephone: +44 20 7636 8636
Fax +44 20 7299 4720
Email: mark.rowland@lshtm.ac.uk

And

Host Country Partners

National Institute for Medical Research
PO Box 9653
Dar es Salaam
Tanzania
Tel: 255 22 2121400/390
Fax: 255 22 2121380/2121360
E-mail: headquarters@nimr.or.tz

Kilimanjaro Christian Medical College

Sokoine Road
Moshi
Tanzania
Tel: 255 272750737
Fax: 255 272836975
Email: fwmosha@gmail.com

Abbreviations

ACT	Artemisinin-based Combination Therapy
COSTECH	Tanzania Commission for Science and Technology
CRERC	Kilimanjaro Christian Medical College Research and Ethics Review Committee
DDT	Dichloro-diphenyl-trichloroethane
DMO	District Medical Officer
DSS	Demographic Surveillance System
EC	Ethics Committee
EIR	Entomological Inoculation Rate
ELISA	Enzyme-Linked Immuno Sorbent Assay
FGD	Focus Group Discussion
GPS	Global Positioning System
Hb	Haemoglobin
HH	House Hold
HRCD	Health Research Challenge for Delivery
ICT	Immuno-chromatic Test
IRS	Indoor Residual Spraying
IVCC	Innovative Vector Control Consortium
JMP	Joint Malaria Programme
KCMC	Kilimanjaro Christian Medical College
LLIN	Long-Lasting Insecticidal Net
LSHTM	London School of Hygiene and Tropical Medicine
LT	Light Traps
MCDC	Malaria Capacity Development Consortium
MRCC	Medical Research Coordinating Committee
MoH	Ministry of Health and Social Welfare
NGO	Non-Governmental Organisation
NIMR	National Institute for Medical Research
NMCP	National Malaria Control Programme
PAMVERC	Pan-African Malaria Vector Research Consortium
PCR	Polymerase Chain Reaction
PHC	Primary Health Care
PI	Principal Investigator
PMI	President’s Malaria Initiative
PMP	Performance Monitoring Plan
PMR	Performance Monitoring Report
PRISM	Pyrethroid and Repellent Insecticide-treated Sheets against Malaria
RDTs	Rapid Diagnostic Tests
RFA	Request for Application
SOP	Standard Operating Procedure
TPRI	Tropical Pesticides Research Institute
TSC	Trial Steering Committee
UCC	Universal Coverage Campaign
URC	University Research Co., LLC
USAID	United States Agency for International Development
WHOPES	World Health Organisation Pesticide Evaluation Scheme

Table of Contents

EXECUTIVE SUMMARY 4
TECHNICAL APPLICATION 4
1. Background 4
2. Research Plan 5
2.1. Objectives 5
2.2. Basic Study Design: 5
2.3. Outcomes: 6
2.4. Hypothesis 7
2.5. Data analysis 7
2.6. Sample Size Estimations 7
2.7. New incidence indicator 9
2.8. Study Area 10
2.9. Co-ordination Committees 15
3. Research activities under the work plan 15
3.1. Mapping of study area 15
3.2. Cluster identification 15
3.3. Cross-Sectional Surveys 17
3.3.1. Overview 17
3.3.2. Pilot Cross sectional survey 18
3.3.3. Baseline and Post intervention surveys 19
3.4. Incidence 20
3.5. Entomology 21
3.5.1. Objectives of the entomological monitoring: 21
3.5.2. Main entomological activity 21
3.5.3. Additional entomological activities 22
3.5.4. Laboratory analysis 22
3.6. Interventions 23
3.7. Monitoring of interventions including sociological data 23
3.8. Data collection and analysis 24
3.9. Ethical considerations 24
3.10. Environmental Compliance standards 24
4. Work Plan 25

EXECUTIVE SUMMARY

The proposed project addresses a critical research question (objective 2 of USAID/URC Request for Application) concerning the operational use of Long Lasting Insecticidal Nets (LLINs) and Indoor Residual Spraying (IRS), namely: Can LLINs sustain transmission reduction gains made by IRS following withdrawal of IRS?

The alliance between LSHTM (UK), NIMR (Tanzania) and KCMC (Tanzania) brings scientists with expertise in vector control research unmatched in Tanzania, encompassing mosquito vector behaviour and ecology, epidemiology, LLIN and IRS coverage and usage, and community-based field trials.

Muleba is an area that was holoendemic for *Plasmodium falciparum* malaria before PMI IRS intervention in 2007. PMI has been conducting IRS in Muleba for the past three years with a single pyrethroid spray round annually. This IRS intervention has already reduced malaria prevalence significantly. Therefore, the setting in Muleba is ideal for research question objective 2. We aim to evaluate the scaling-up LLIN coverage across the whole of the study area and withdrawing IRS in one study arm whilst maintaining a single IRS round in the other arm. The study will run for two years.

The trial design will involve randomised selection of stratified clusters for 2 study arms. The primary outcome measure will be prevalence of parasitaemia and anaemia in children aged 0.5-10 years, measured in cross sectional surveys.Secondary outcomes will include malaria transmission as measured by entomological inoculation rates (EIR) of the mosquito vector species and serological prevalence of malaria, as well as user acceptability of LLINs compared with IRS. It is hoped that a measure of incidence can be introduced, based on clinical malaria diagnosed using RDTs in the health facilities of the district.

The study location and the proposed trial design will provide PMI and National Malaria Control Programme with valuable answers to the key objectives. Findings from this study are expected to inform decision making so that resource utilization can be optimised.

TECHNICAL APPLICATION

1. Background

In Tanzania the 2010 program of the National Malaria Control Programme (NMCP), in collaboration with several international donors (including President Malaria Iinitiative), will scale up LLIN distribution under the Universal Coverage Campaign (UCC) and spray IRS insecticides in selected regions with the highest malaria prevalence (USAID, 2009). The universal coverage campaign is scheduled to take place in Muleba district at the end of March 2011. Historically control programs have focussed either on IRS or ITN/LLINs. This trend is changing with ambitions towards elimination and we are to see implementation of both IRS and LLIN in several African countries under PMI. One potential control strategy involves using IRS in areas of high transmission to first reduce transmission to a low level before using LLINs to maintain the low

parasite rates while withdrawing IRS. It is essential to conduct cluster randomised trials to determine whether LLINs at high coverage can provide a non inferior replacement of IRS after several cycles of the latter.

The results of the proposed trial will help inform PMI, the Tanzanian NMCP and other African Departments of Health of the potential benefits and detrimental effects of maintaining or introducing IRS alongside scaling up of LLIN distribution. The results will be beneficial to the study communities and similar populations in Tanzania and elsewhere. The study will also provide evidence regarding the cost and effectiveness of 1 round of IRS per year in comparison to scaling up LLINs.

The National Institute for Medical Research (NIMR) and Kilimanjaro Christian Medical Centre (KCMC) are major malaria research organisations in Tanzania. They have a long history of working with the London School of Hygiene & Tropical Medicine (LSHTM) on malaria research under the umbrella of the Pan African Malaria Vector Control Research Consortium (PAMVERC). More recently LSHTM, NIMR and KCMC have evaluated different types of LLIN on behalf of the WHO Pesticide Evaluation Scheme (WHOPES) and the manufacturing industry. They also constitute the field evaluation wing of the Innovative Vector Control Consortium (IVCC) whose purpose is to work with industry to develop new vector control tools such as combination nets and long lasting IRS formulations. Thus the three partners; LSHTM, NIMR and KCMC, are pre-eminent in the development, implementation and evaluation of IRS and LLINs, and with their long history of collaboration form the ideal technical and managerial alliance to conduct the research planned for PMI and advise on future practice.

2. Research Plan

2.1. Objectives

The aim of this research is to determine whether it is necessary to maintain IRS once malaria transmission has been reduced or whether following the scaling-up of LLINs the IRS can be withdrawn and low transmission rates can be maintained equally well with LLINs alone. It will specifically address objective 2 of the RFA:

“Objective 2. In areas where one or more rounds of IRS have been carried out and malaria transmission has been reduced, can LLINs be used to sustain the transmission reduction gains made by IRS following the withdrawal of IRS?”

2.2. Basic Study Design:

The study objective will be addressed as a two-arm cluster-randomised intervention trial.

Study arms:

Study arm A. Yr. 1: One round per annum of high coverage IRS and high coverage LLIN,
Yr. 2: One round per annum of high coverage IRS and high coverage LLIN.
Study arm B. Yr. 1: One round per annum of high coverage IRS and high coverage LLIN,
Yr. 2 – No IRS and high coverage LLIN.

The null hypothesis would be that the intervention arm in which IRS was withdrawn (Study arm B) is inferior to the reference arm in which IRS is retained (Study arm B). The alternative hypothesis is that the LLIN only arm (Study arm B) tis non-inferior to the LLIN plus IRS study arm (A), as demonstrated by a difference in prevalence of infection in the two study arms to be no more than a pre-specified margin. If IRS is eventually withdrawn programmatically from areas currently given IRS and LLIN universal coverage, this study would provide assurance that it would be safe to do so without endangering a return to higher transmission levels.

2.3. Outcomes:

Primary outcomes:

1. Prevalence of malaria infection in children 0.5-10 years
2. Mean haemoglobin (g/dL) in children under 5 years.

Secondary outcomes:

1. Incidence of confirmed malaria episodes in children and adults through passive case detection at district health facilities
2. Sero-conversion rates
3. Entomological Inoculation Rate (EIR) for each mosquito vector species.
4. Relative population density for each mosquito vector species
5. Usage and perception of existing LLIN in baseline year
6. Evaluation of the Universal Coverage Campaign of LLIN
7. Perception and acceptance of IRS in baseline year
8. Usage and perception of LLIN in an environment of reduced IRS.
9. Detection and monitoring of resistance markers including *kdr*

Previously, in the absence of hard epidemiological data, we were unable to decide whether prevalence or incidence should be selected as our primary indicator. We think that an incidence indicator may be feasible provided we can enlist the commitment district health authorities and health centre staff to the new concept. The new concept is outlined in section 2.7. Parasite prevalence remains the primary indicator but until we have an indication of baseline prevalence we cannot estimate the number of clusters that need to be enrolled to demonstrate equivalence. Based on recent calculations it seems likely that around 30 clusters (15 clusters per study arm) will be required. One of our first activities will be to estimate prevalence in around 50 clusters in the study area. This will allow a more accurate estimation of the required sample size. We will then enrol the required number of clusters into the primary study, selecting those with the highest prevalence.

2.4. Hypothesis

This trial will address the following question: In a situation where IRS is thought to have been successfully deployed to reduce malaria transmission, can high coverage LLINs be implemented as a non-inferior alternative to continued IRS plus high coverage LLINs and achieve the same level of malaria control. In terms of study design the trial will, after a baseline year of IRS and high coverage of LLIN in both arms, compare high LLIN coverage along with continued use of IRS in one arm to high LLIN coverage alone in the other.

In year one (baseline) the two study arms both receive one round of high coverage IRS in January-February 2011 and Universal Coverage of LLIN at the end of March 2011. Coverage of both will be assessed. In year two (intervention year) the reference (control) study arm A continues with one round of high coverage IRS in September 2011 and high coverage of LLIN (from the distribution in March), whilst in the intervention arm (B) there will be continued high coverage of LLINs and IRS is withdrawn. The intervention arm should be no less effective than the reference arm.

2.5. Data analysis

Since compliance with LLIN or LLIN plus IRS allocation may not be fully adhered to, both intention to treat and per protocol analysis will be performed. To do this, household ownership and usage of LLINs and whether the house was sprayed will be determined for each individual whose infection status is measured, in each survey. Data analysis will be based on methods that make allowance for between-cluster variation (intra-cluster correlation) of responses. Data analysis will be carried out using both cluster level approaches and individual level regression approaches using random effects models. Point estimates and confidence intervals of rate ratios of malaria episodes (odds ratios of infection prevalence and anaemia) in intervention arms, relative to the reference arm, will in the first instance be based on cluster summaries. Poisson regression will be used to estimate intervention effects on malaria incidence rates and logistic regression models will be used to estimate intervention effects on prevalence of infection and prevalence of anaemia, adjusted for individual and cluster specific confounders, using random effects models.[1]

By including vector species (*Anopheles gambiae s.s.*, *An.arabiensis* and *An.funestus*) as a cluster level co-variate, it may be possible to investigate the effect of vector species on vector control effectiveness.

2.6. Sample Size Estimations

The sample size is based on the principles cluster randomised trials[1] using the margin of difference of prevalence of infection between the IRS plus LLIN and the LLIN alone arms. As the trial is intended to show non-inferiority between the LLIN and IRS study arms, power calculations have been based on an observable margin of difference between these two study arms. The cost considerations require this margin of difference to be 50% or more. It is appreciated that a 50%

[1] Hayes RJ and Moulton LH. Cluster randomised trials. Chapman Hall/CRC. New York, 2009.

relative difference between study arms may be unacceptably large when prevalence is high. For example when prevalence it high an absolute margin of difference of 20% would not constitute meaningful equivalence, whereas 2% or 3% difference may well be considered equivalent, even if this difference constitutes a relative difference of 50% or 75% respectively, when true prevalence is only 4%. Prohibitively large numbers of clusters would be required to determine non-inferiority within a smaller margin of relative difference between study arms. The baseline malaria indicator survey will be used to estimate the current prevalence of infection in the study area.

Table 1 shows the number of clusters per study arm required in superiority and non-inferiority trials respectively, for different scenarios of relative differences in prevalence of between study arms, levels of baseline prevalence of 10%, 8% and 4% respectively, and for between cluster coefficients of variation of 0.1, 0.2 and 0.3 and one scenario of 0.6. The sample size per cluster is 100 throughout; power is set at 80% and significance at 5%. The scenarios have been chosen for absolute differences between study arms of no more than 5%, which has been assumed to be the largest margin of difference that would be meaningful in a non-inferiority study, even if it represents a relative difference of 50% (or more).

We expect that the baseline survey may show prevalence to be quite low, and have therefore included scenarios where baseline prevalence is 10% or lower, and where one might want to show non-inferiority as ruling out a LLIN alone arm inferiority of more than a few percent prevalence above the IRS plus LLIN arm.

Table 1. Sample size scenarios

Prevalence of infection[2], %	Relative difference/ margin (absolute difference) between study arms	Between cluster coefficient of variation	Persons surveyed per cluster	Clusters per arm required in SUPERIORITY trial	Clusters per arm required in NON INFERIORITY TRIAL
10	50%(5)	0.1	100	6	6
10	25% (2.5)	0.1	100	23	21
8	50% (4)	0.1	100	7	8
8	25%(2)	0.1	100	29	26
10	50%(5)	0.2	100	7	8
10	25%(2.5)	0.2	100	29	27
8	50%(4)	0.2	100	9	9
8	25%(2)	0.2	100	35	32
10	50%(5)	0.3	100	9	10
10	25%(2.5)	0.3	100	39	37
8	50%(4)	0.3	100	11	12
8	25%(2)	0.3	100	45	42
4	75%(3)	0.3	100	7	9
4	75%(3)	0.6	100	11	15

2.7. New incidence indicator

The establishing of incidence through passive case detection in the existing primary health care (PHC) system is feasible and has the advantage of building upon the existing health care structures. The current PHC system based on government and private (e.g. missionary) funded health facilities is staffed by committed and experienced health care workers. Such facilities are in place throughout the district and their exact locations have recently been mapped by project staff (Fig 3). What is currently lacking is the means to accurately diagnose malaria and to manage it properly. Some facilities diagnose clinically, some through microscopy, and all prefer RDTs when they are available. Because of frequent RDT stock-outs it is impossible to assess the true burden of malaria or show if it is declining as a result of the PMI IRS programme. If RDTs and ACTs were to be made available throughout the year the basic means to diagnose malaria would be in place. If the registration system were then to be modified to record each patient's age and hamlet, village and ward of residence the basic system needed to determine passive case incidence and the impact of the PMI control programme would be established. Three outcome variables would emerge from such a system: 1. the number of malaria cases per year by cluster and age group, 2. the ratio of malaria to other causes of febrile illness (equivalent to the indicator 'slide positivity rate' in countries where microscopy is conventionally used) and 3. incidence rates among registered

[2] Prevalence in control arm for superiority study; true prevalence in non-inferiority study

families from the clusters. For the duration of the project copies of the records would need to be collected from the health centres on a monthly basis and then the data would need to be entered into an access database to allow analysis.

Establishing such a system has many benefits not just to the project but to PMI's greater aims:

1. It provides a role for the general health services and the District Medical Officer in monitoring progress of the malaria control programme.
2. It improves the quality of the PHC system to improve diagnosis and treatment of malaria.
3. If PMI were to scale down its control programme and withdraw from the area, a system would be in place to provide continued management of malaria, possibly reducing any risk of re-emergence and could provide a warning system if malaria transmission started to increase.

Establishing such a system cannot be done by the Project itself. It requires commitment, partnership and coordination between the Project, PMI, USAID, NMCP, DMO, general health service facilities. There is a clear role for PMI in making sure that adequate resources (RDTs and ACTs in particular) are made available to ensure year round diagnosis and treatment ad libitum, and to ensure that its partners (RTI and local PMI) commit to the endeavour. Coordination mechanisms need to be put in place. All partners need to be fully committed. If all goes to plan a model for provision of diagnosis and treatment will be established that can then be replicated in other areas.

The inspiration for this system arises in part from the study done by Fabrizio Molteni and colleagues in RTI and PMI/CDC in which RDTs were supplied to 17 health facilities in IRS and non-IRS areas of Kagera, where it was shown that positivity rates fell from 9.6% to 2.8% in control and sprayed areas respectively (Molteni et al. 2010). Similar approaches to monitoring incidence have been established in other countries and are feasible in Muleba.

2.8. Study Area

The trial will be carried out in rural villages in Muleba district (1^0, 45'N, 31^0, 40'E), in Kagera region of north-west Tanzania (fig. 1). Muleba District covers an area of 10,739 km^2, of which Lake Victoria is 62%. Most of the district lies at 1200-1500m above sea level. Administratively the district has 31 wards, 134 villages and a population of 425,172 people (URT, 2003) with 85,035 (20%) children <5 years old. The district has 36 health facilities comprising 3 hospitals (Rubya, Kagondo and Ndolage), 4 health centres and 29 dispensaries. Parasitaemia and anemia prevalence ranges widely between villages. *An. funestus* is the predominant vector in many villages within the district, representing 58-66% of the total catch (NIMR, unpublished data).

The district was chosen as a launch site for PMI-funded spray operations in 2007. In 2009 PMI supported three rounds of IRS in Muleba district, achieving over 90% coverage (fig. 2).

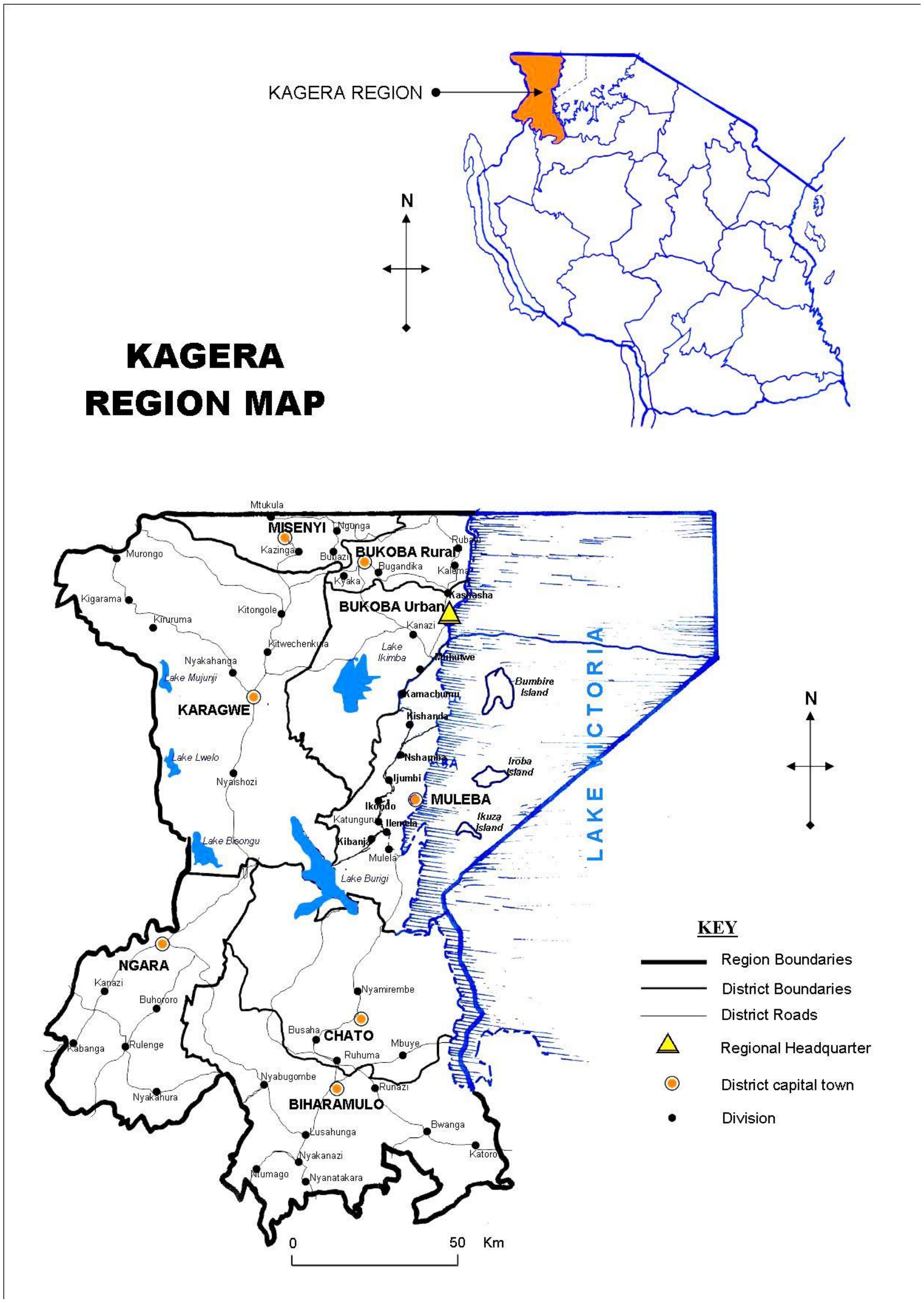

Figure 1. Kagera Regional map with administrative boundaries

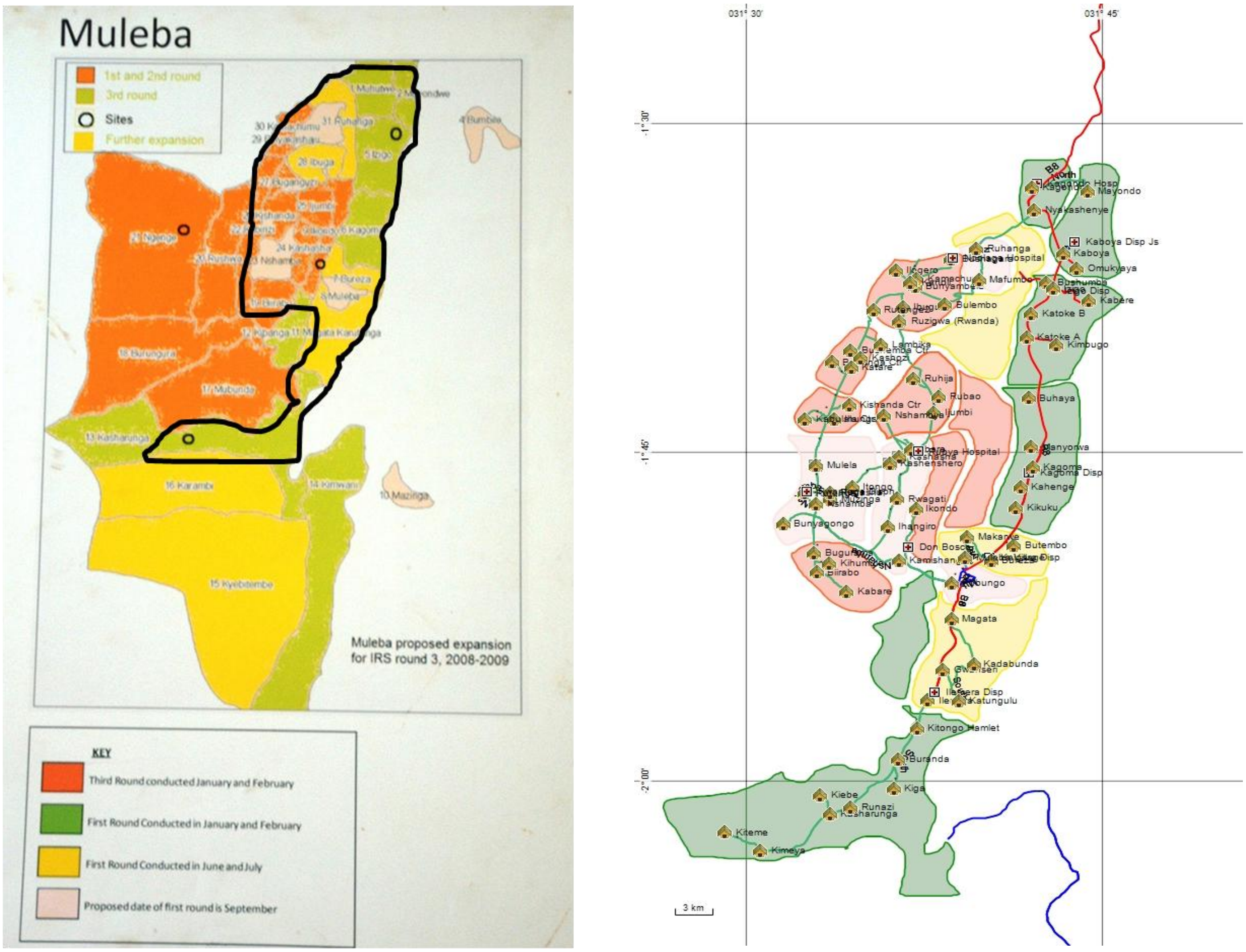

Figure 2. Left = Spray coverage in Muleba wards (image courtesy of RTI). Black line demarcates study area for PAMVERC research project. Right = clusters geo-located by the PAMVERC project during initial site visits. Coloured blocks correspond with alleged spray rounds.

The district has two rainy seasons March to June and September to December, but in the last ten years January and February have also experienced considerable rainfall (figure 3).

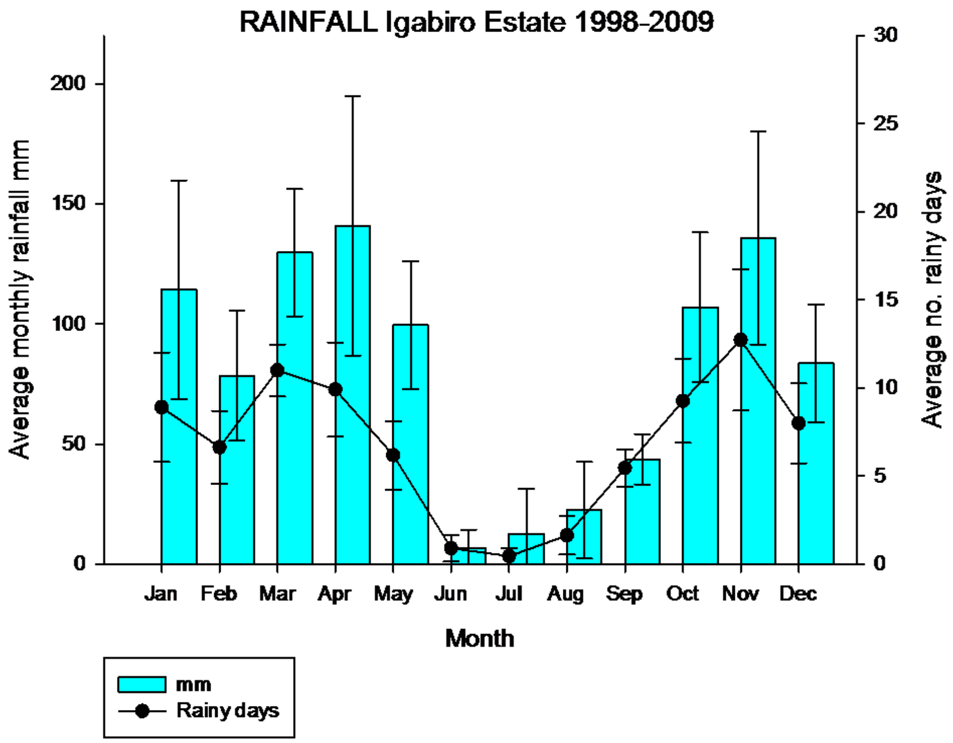

Figure 3. Average monthly rainfall and the average number of rainy days (error bars = 95% C.I.) at Igabiro Estate, Nshamba Division, Muleba, between 1998-2009.

Pre-IRS parasite prevalence ranged from 10-31% in selected villages 6 months post-IRS a two-fold reduction of malaria prevalence to an average of 9.8% was observed (NIMR survey, Kinug'hi et al., unpublished, fig.4). RTI have also assessed the effectiveness of the IRS campaign. They report an overall RDT positivity rate from 9-month old children and pregnant women of 2.8% (N=3884) in villages that received IRS versus 9.6% (N=2916) in unsprayed villages (Molteni et al., personal communication). The Muleba health facility (HF) records seem to provide further evidence that malaria is declining, though incidence cannot be accurately determined because the majority of these records constitute clinically diagnosed rather than microscopy diagnosed cases and the majority of these will not be true malaria. In 2007 there were 83,046 suspected malaria cases reported from 25 HF, in 2008 this stood at 82,978 cases (from 29 HF) and to 64,645 in 2009 (29 HF).

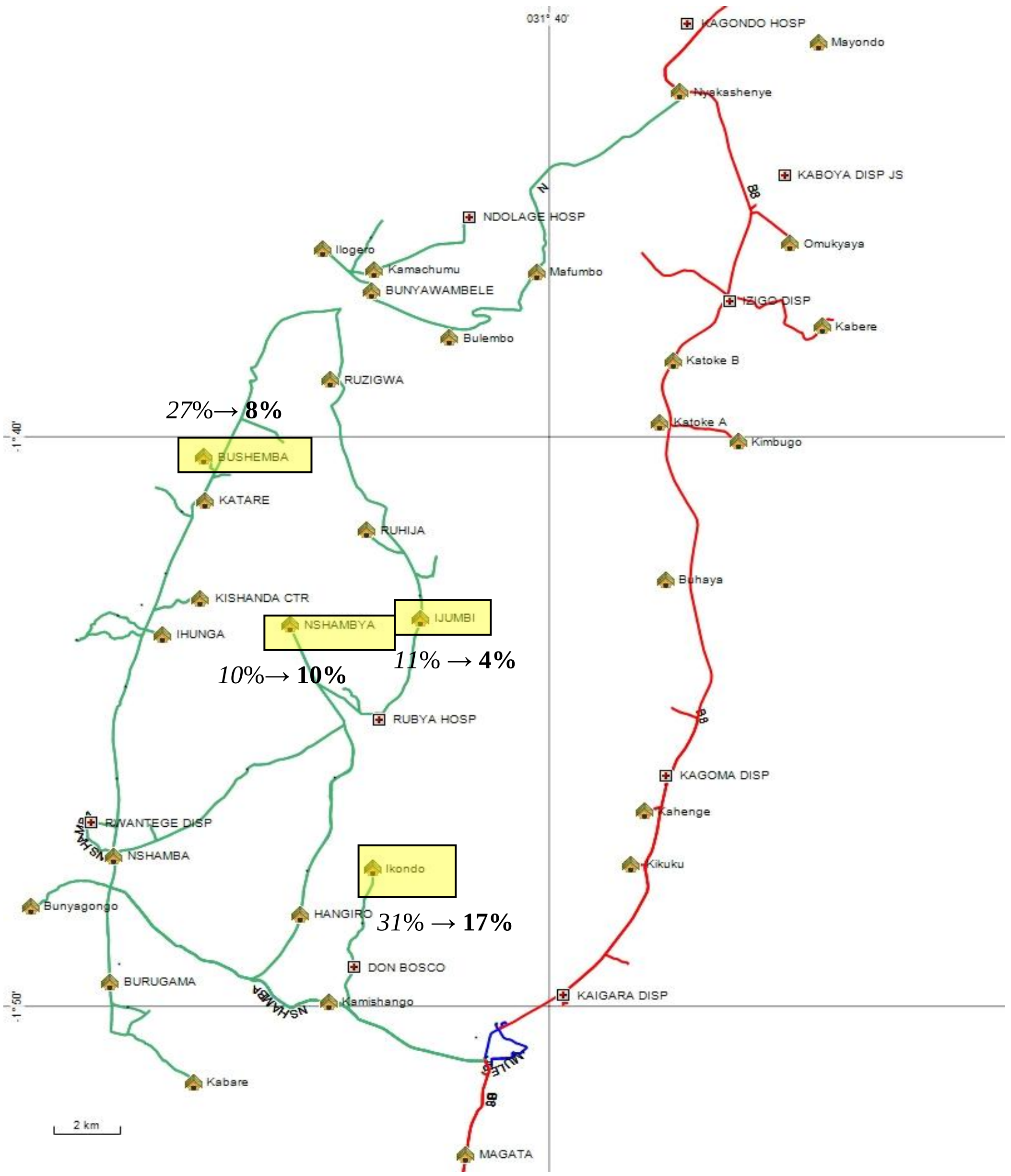

Figure 4. Close-up map section showing NIMR prevalence study villages highlighted in yellow with *pre*- and **post-**IRS malaria prevalence % values. Also shown are the locations of major health facilities in the study area.

Bed net coverage has increased during the same period. 34% of children and 29% pregnant women were sleeping under bed nets (untreated, ITN, and LLIN) in Kagera Region, with a marked urban-rural skew in 2009 (Widmar et al., 2009). RTI reported that now there are ~60% of pregnant women and under-fives sleeping under an LLIN, with 40% coverage overall (Molteni, personal communication).

One of the host country partners, NIMR, has a significant presence in Muleba district and has been directly involved with monitoring the PMI IRS spray programme through follow-up entomological

surveys and the monitoring of mosquito resistance in the district. It is hoped to integrate their involvement within the Project.

LSHTM and KCMC also have a long term presence there at a research station in Mwanza, a centre for research on STIs and HIV. Through these offices the NIMR, LSHTM and KCMC malaria groups can readily expand its geographic range to Kagera region.

2.9. Co-ordination Committees

The research project involves several stakeholders and interested parties commitment of which is essential to success. The Project is working closely with RTI to co-ordinate spraying of study villages, NMCP which is responsible for all malaria control activities in Tanzania, MEDA/World Vision/PSI who are involved in LLIN distribution. Regular communication is necessary with the district medical representatives to link up with dispensaries, enable timely and adequate provision of RDTs and ACTs, and working with nurses and clinicians. Two co-ordination committees will be established. The first will include senior representatives of WHO, NMCP, RTI and PMI/USAID as a way of informing on project progress in Dar es Salaam. The second committee will be at the regional level involving the district medical officer, malaria focal person, RTI representatives.

3. Research activities under the work plan

3.1. Mapping of study area

During initial visits we identified and geo-located 75 villages within the district that were accessible and within a travelling time from PAMVERC office in Muleba town of less than 45 min. The study area has since been extended to cover a total of 113 villages across 29 wards. These villages lie in an area of approximately 2000km^2 extending 70km North to South and 68km East to West, at an elevation ranging between 1110 to 1640m above sea level. The total population of the study area is around 68,000 houses.

Every building of each hamlet mapped using a Global Positioning System handled units (GPS) (Garmin Legend e-trex) and ExpertGPS v3.8 (TopoGrafix) software. Boundary of each hamlet, health facilities and other landmark are also geo-located (figure 5).

3.2. Cluster identification

With the maps it becomes possible to identify clusters to use in the cross sectional prevalence survey (figure 5 and 6). Each cluster will be comprised of at least one village, because the village is the smallest unit we can refer to when directing the different vector control measures (IRS and LLIN distribution) during our intervention year. By dragging concentric circles over the maps we can identify suitable clusters of one or more villages . Each cluster includes:

a. A core sampling area of approximately 1 km radius including a minimum 200 household where the cross sectional surveys and entomological monitoring are conducted
b. A buffer zone of at least 1 km surrounding the core sampling area, where hamlets will receive the same intervention as the survey area.

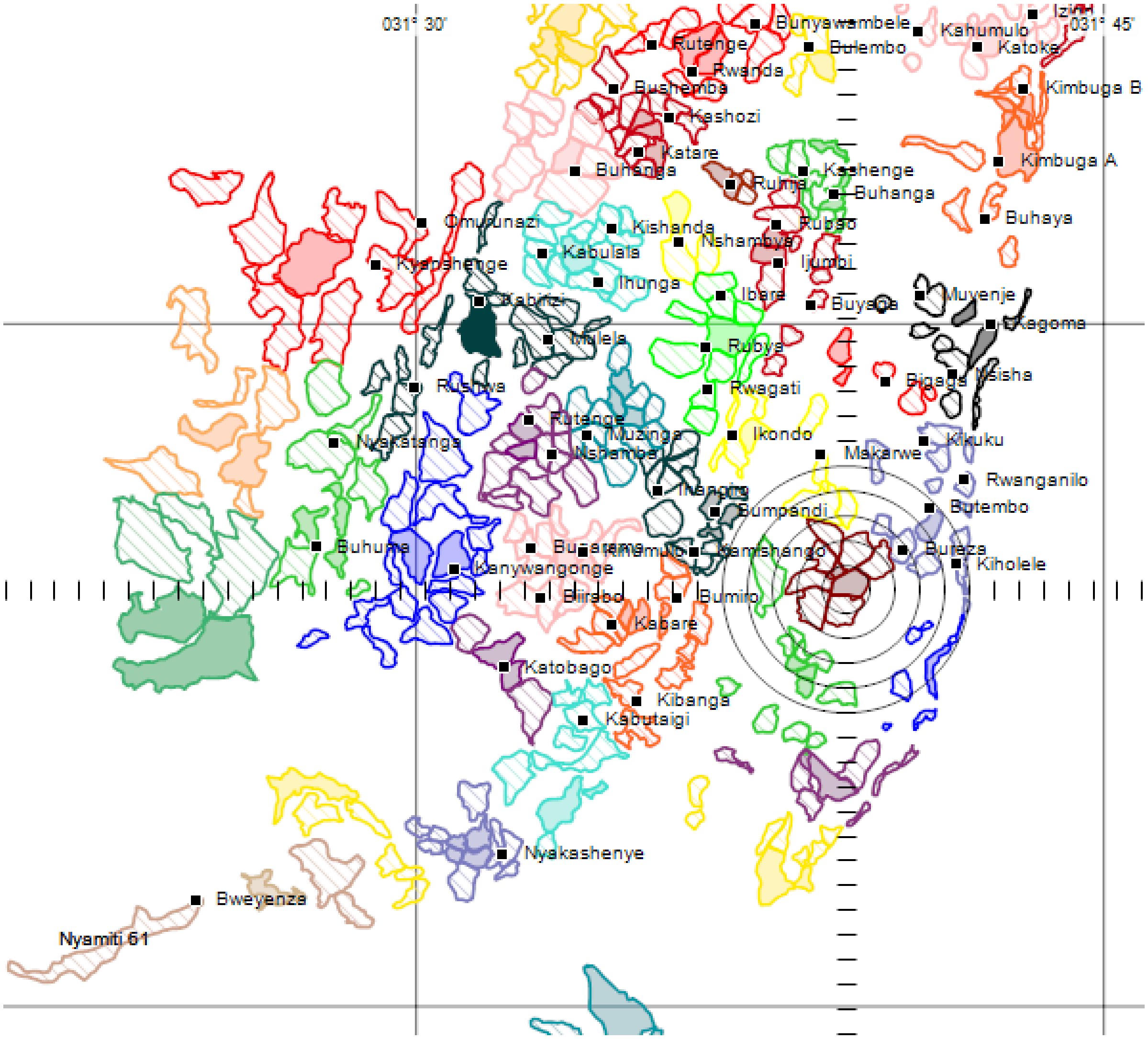

Figure 1. Hamlet distribution (▪ = village centre) in the study area. Each shape represents a Hamlet. Clusters are identified with a same colour and the plain shapes show the core sampling areas within the cluster where surveys are going to take place. In the example circled in red the cluster is formed by 9 hamlets, 2 are part of the core sampling area and the 7 other are in the buffer zone.

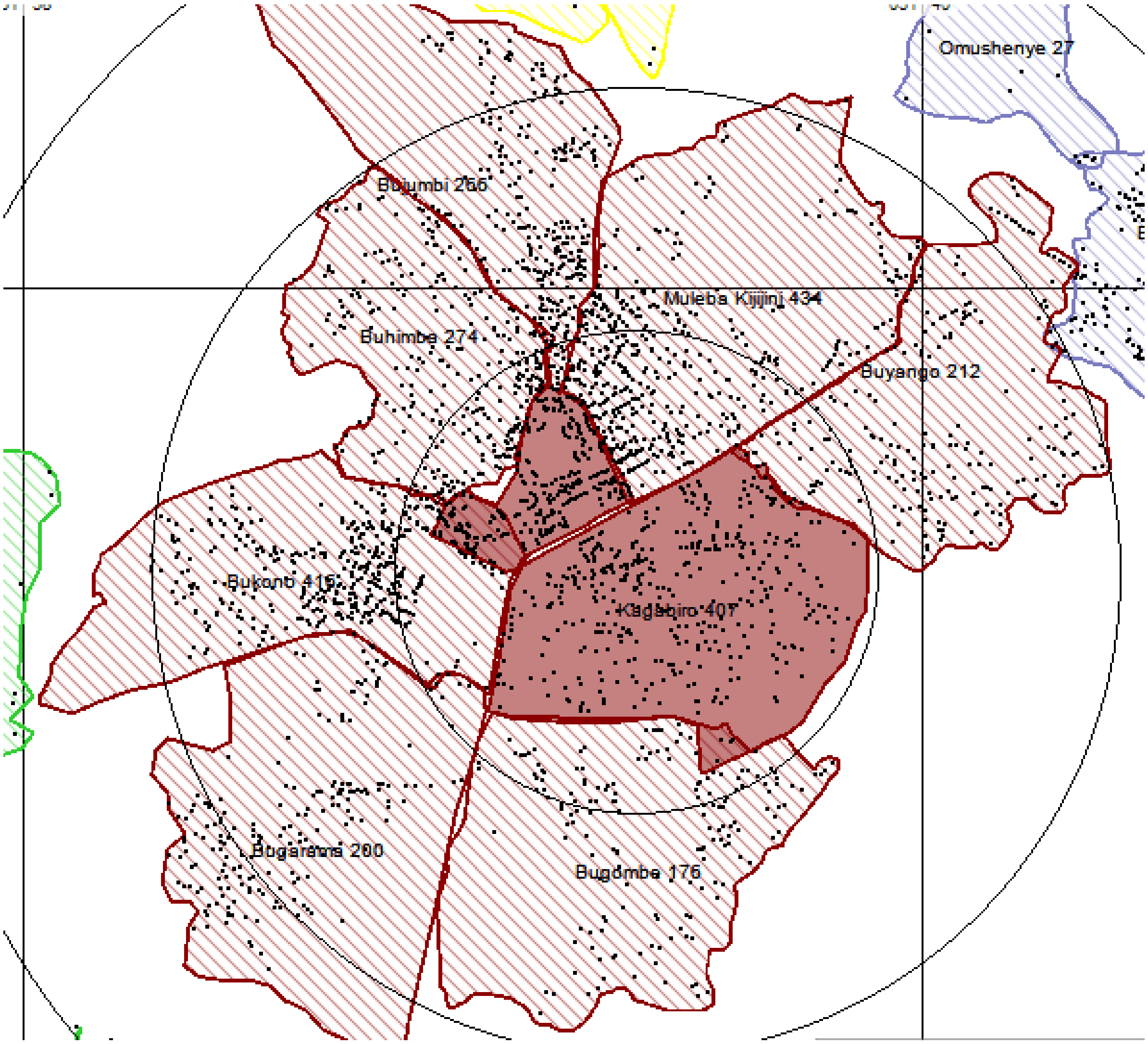

Figure 6. Detailed map of one cluster. Small black dot represent the houses.

3.3. Cross-Sectional Surveys

3.3.1. Overview

There will be four cross-sectional surveys during the study period;

- Pilot cross sectional survey– February - March 2011
- Baseline cross sectional survey– June 2011

Intervention: Withdrawal of spraying in one arm September 2011

- Post Intervention Survey A– February - March 2012, 5 months after the intervention
- Post Intervention Survey B– June 2012, 8 months after the intervention

Each survey will include two components; 1) a household component and 2) a parasitological component. The household component will be conducted in 100 houses selected at random from the core sampling area (figure 7). A questionnaire will be administered to obtain information on possible

risk factors for malaria and a maximum of two children from eligible household will be selected. Children will be sent to a central point the following day for the parasitological testing.

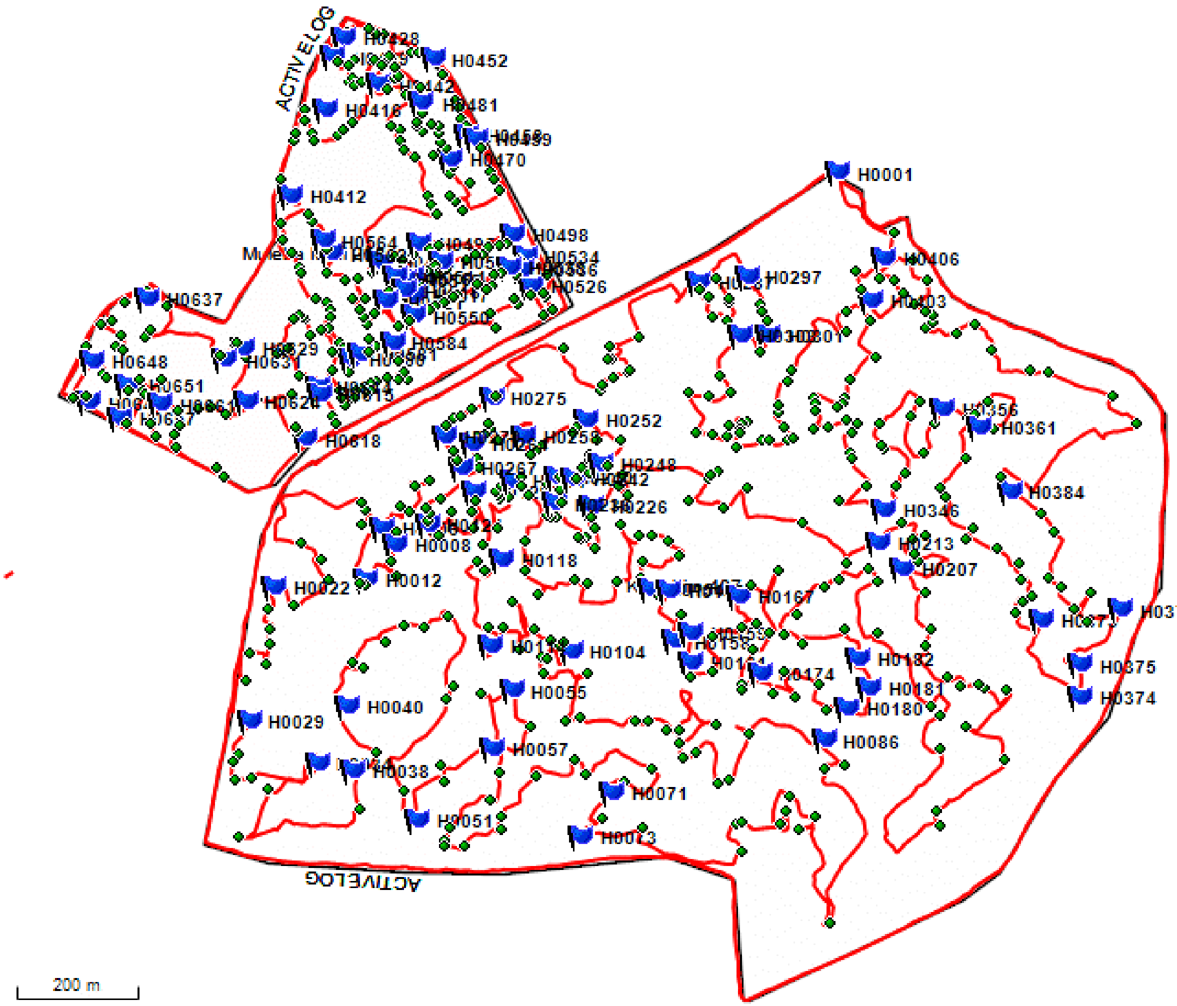

Figure 7: Detailed map of the survey area of one cluster. Blue flag represent the houses that have been selected for the survey and need to be visited. Green dot indicate the other houses.

3.3.2. Pilot Cross sectional survey

Objectives of the Pilot

1. To estimate the malaria prevalence rate in each of the 51 clusters,
2. To determine the cluster sample size for the main study
3. To select the clusters with the highest prevalence rates for inclusion into the main study
4. To pilot the process and tools to find any problems
5. Determine the age distribution of malaria parasite prevalence and guide the age ranges to be included in the following surveys

Household component

One hundred households are randomly selected from the core sampling area of every cluster. In each house a maximum of two children between 6 months and 14 years will be selected. This will yield approximately 100 children allowing for refusals and non-response. A total of 5000 children are expected to be surveyed. Paper questionnaires are used and are double entered by two data-entry clerks into an access database.

Parasitological component
All selected children have their temperature taken, and are tested for malaria parasites using RDTs, thick and thin blood smears. When RDT is positive free treatment for malaria will be provided. Children with severe malaria or any other disease that can't be treated by the team will be referred to the nearest health facilities.

The slides will be fixed, stained and checked for quality in Muleba by a laboratory technician. Slides are double read, blind by two experienced microscopists. Slides are declared negative after reading 200 fields. If positive, parasite species are reported (including mixed infections) and are counted against 200 white blood cells. Gametocytes are recorded for Plasmodium falciparum.

Selecting the clusters for baseline and post interventions surveys
The selection of the determined number of clusters for the main study will be based on the following criteria determined from the pilot cross-sectional survey:

1. Clusters with the highest estimated malaria prevalence will be included.
2. Exclusion of all clusters that include any areas that have not been sprayed in the last spray round.

Allocation to the two arms will be at random, stratified by malaria prevalence and population size as determined by the pilot study.

3.3.3. Baseline and post intervention surveys

These surveys will follow the same process than the pilot survey. However the questionnaire will be more detailed and additional tests (haemoglobin, serology, PRC) included in the parasitological component. The age distribution of the individuals to be included in these surveys will be based on the finding of the pilot study result.

Objectives of the baseline survey

1. To determine if the two arms are similar in terms of malaria indicators
2. To determine the coverage of LLINs and evaluate the universal coverage campaign
3. To evaluate the usage and perception of existing LLIN in baseline year
4. To evaluate perception of IRS in baseline year
5. To determine the risk factors for malaria
6. To determine the effect of LLIN integrity on malaria prevalence

Objectives of the post-intervention surveys
Compare the two study arms in terms of:

1. Malaria prevalence
2. Mean haemoglobin (g/dL) in children under 5 years old
3. Sero-conversion rates

4. Entomological Inoculation Rate (EIR) for each mosquito vector species.
5. Relative population density for each mosquito vector species

Evaluate:

6. Usage and perception of LLIN in an environment of reduced IRS.
7. Coverage and perception of IRS and the withdrawal of IRS
8. To determine the effect of LLIN integrity on malaria prevalence

<u>Household Survey – modifications from the pilot</u>
The survey will include additional information on, educational status and occupation, social economic status (SES) and house structure. Additional question on nets will be elaborate to evaluate the LLIN distribution campaign process of March 2011, source of net and net usage and reason of non usage. The IRS section will also include in-depth information on coverage and perception and acceptance of IRS.

It is planned to use a Personal Digital Assistant (PDA) for the household questionnaire during baseline and post interventions cross sectional surveys.

<u>Parasitological Survey– modifications from the pilot</u>
Additional data and test will be performed:

- History of fever in the last 48 hours will be recorded
- Only individuals with current or history of fever in the last 48 hours will be tested for malaria with an RDT.
- ***PCR.*** A blood spot on filter paper will be collected for PCR determination of sub-patent infections since prevalence is low. This is to be done for all individuals. The presence of parasite may have been much reduced as a result of the previous IRS campaigns.
- ***Force of infection as determined using serology:*** A blood spot on filter paper will be collected for serological estimation of antibodies to malaria. Which individuals will be sampled for serology will be determined by the pilot and through discussion with serology specialists. As vector control has been ongoing since 2007 serology will show us the change in the force of infection over time since IRS intervention began. The purpose is to determine whether malaria prevalence fell around the time IRS spraying began.
- ***Prevalence of anaemia:*** Haemoglobin will be measured using Hemocue and the prevalence of anaemia (<8g Hb/dL) will be calculated. Body weight, height and upper mid-arm circumference will be measured to allow adjustment for possible malnutrition. Iron supplementation will be given to any child with anaemia (Hb<8 g/dL).

3.4. Incidence

The plans for establishing a new incidence indicator based on passive case detection through government, NGO and faith group health facilities was described in section 2.7. Consensus and new commitment would be required by the Ministry of Health, district health services, PMI, RTI among others. The facilities to include would be those serving the study area catchment clusters. Sufficient RDTs and ACTs need to be made available. That may require repartitioning of government or PMI resources from other districts and it is presently unclear whether there is sufficient budget flexibility to establish the new system. Ideally provision should be made throughout the study periods (baseline and intervention years), but if this is not feasible provision should assigned to the peak malaria seasons

during the two years. To use clinical incidence data as a study outcome requires that the cluster in which each patient lives is known. It is intended that this will be done by issuing identification cards to participating households in each cluster, and asking householders to bring these to the clinic with them, so that this can be noted in the register. This will require the necessary collaboration from clinics in the study area. If this system is not feasible we shall encourage clinics to ask information about the village of residence of all outpatients with suspected malaria (prior to RDT) and analyse the data accordingly.

3.5. Entomology

3.5.1. Objectives of the entomological monitoring:

The main objectives of entomological monitoring are;

a. To determine abundance and relative proportion of *Anopheles gambiae* and *An. funestus* sibling species between study arms
b. To record differences in EIR between study arms

Secondary objectives

c. To monitor resistance in vector and nuisance biting mosquitoes.
d. To assess insecticide longevity
e. To assess behavioural change

3.5.2. Main entomological activity

Monitoring of mosquito population density, human biting rates, entomological inoculation rate
Mosquito control through IRS has been ongoing in Muleba since 2007 and is likely to have had a significant impact on mosquito populations. Pre-IRS surveys by NIMR Mwanza showed the presence of both *An. funestus* and *An. gambiae s.l.* The relative composition and abundance of these species is likely to have changed significantly as a result of IRS.

Pilot: stage 2
During stage 2 (pilot) the purpose of mosquito trapping is to guide our subsequent entomology protocol during stage 3 (baseline) and stage 4 (intervention years). 200 trap nights and space spray catches in 200 additional houses will be conducted during the pilot to determine the abundance and relative proportion of vector species. The 8 houses will be selected at random in the core sampling area of each cluster. For each of the selected houses a description of the HH will be done using a questionnaire, including information on the number of inhabitants, type of house (wall, roof, number of rooms, number of sleeping places etc.), presence of animals and malaria prevention measure (IRS, net, any others) used by the household members.

Reports from the district indicate a substantial decrease in malaria following IRS and this may have resulted in very low mosquito densities. If mosquito catches are prohibitively low during the pilot survey, longitudinal monitoring during the baseline and intervention period may lack sufficient power. This information will allow us to modify the entomology protocol to be more intensive during certain time periods for specific objectives such as resistance monitoring and

sporozoite infectivity. Relative proportions of *An. funestus* and *An. gambiae s.l.* trapped during the pilot determine which probes are to be ordered for species identification.

Entomological monitoring during stage 3 and 4
Fifteen clusters per study arm will be selected for longitudinal entomological monitoring. Collections are done monthly during one day/night in 10 households in each of the clusters. Collection is done by using the best method, light traps or space spray catches, depending on the result of the pilot. The methods will be similar to those used in the pilot including household questionnaires etc.

This gives an estimate of the number of mosquitoes and any change in the size of the mosquito population in the village due to the IRS and LLIN interventions. Each cluster is trapped once a month. The catches are identified to species, counted and feeding status recorded. Females are stored in individual capsule and kept for sporozoite analysis, for sibling species identification, and kdr testing, males are discarded.

3.5.3. Additional entomological activities

Behavioural change assessment
As vector control activities have been ongoing for several years there is a possibility that indoor resting and biting mosquitoes may have been selected for behavioural modification and outdoor resting/biting may be more common. Sentinel cows and pit trap collections will be used for periodic sampling for evidence of outdoor biting. By using a variety of trapping methods we are able to show changes in distribution, density and behaviour of a range of mosquito vector species that exhibit different behaviour and observe any changes in resting preference over time in response to different IRS frequency and increase in LLINs.

Insecticide residual activity on sprayed walls and LLINs and insecticide resistance
It is important for us to monitor coverage and quality of IRS and LLINs during the intervention year. Pyrethroid detection kits for IRS and LLINs developed by LSHTM will be deployed to verify spray coverage and continuing efficacy of LLINs.

Cone bioassays will be carried out bi-monthly post spraying during the stage IV to determine residual activity of the IRS insecticide. Where possible we shall work in partnership with the entomology unit of NIMR Mwanza, which has been monitoring insecticide performance for RTI, for bioassay of IRS surfaces.

Insecticide resistance in vector and nuisance-biting mosquitoes
Pyrethroid resistance is a particular concern in areas of high selection pressure where pyrethroid IRS and pyrethroid LLINs are used. WHO resistance tests will be carried out once a year using pyrethroid test papers in WHO kits. Percentage mortality, median time to knockdown, and LD50s response to pyrethroids will be measured. Longitudinal sampling of mosquitoes and testing for kdr and other resistance markers will allow detection of resistance and selection in the two study arms.

3.5.4. Laboratory analysis

There are several laboratory techniques that will be important in producing robust entomological outcomes.

Entomology techniques planned for this study include mosquito sibling species identification (*An. gambiae* & *An. funestus* complexes) and investigation of resistance mechanisms (Kdr) through real-time multiplex PCR, sporozoite ELISA and blood-meal identification.

Some of the protocols involving conventional low-throughput systems have been updated for high throughput and cost-effective analysis of samples following the IVCC Vector Population Monitoring Tool.

To fulfil the objectives set out in the workplan initial training will be required, the Liverpool School of Tropical Medicine (LSTM) have agreed to be involved in equipment set-up and protocol training.

3.6. Interventions

During stage 2 in February 2011 an IRS round has been completed in Muleba district including all the selected clusters of our survey area. Another round is planned in September-October 2011 and will target only half of the cluster randomly selected to the IRS plus LLINs study arm.

In March 2011, a LLIN distribution campaign supported by Mennonite Economic Development Associates (MEDA) and funded by Global Fund is scheduled and aims to reach universal coverage. However preliminary information shows a really low bed-net usage, this suggests that an intensive Information Education Communication (IEC) campaign is required to get high usage of LLINs.

Additional net will probably need to be secured to top up the universal coverage campaign and insure that every sleeping place is covered by a LLIN in the survey area. The bed-net coverage given by the pilot survey will help us to estimate the quantity of LLINs needed to reach universal coverage and identify if additional LLINs are required after the Global Fund LLIN distribution of March. LLIN integrity will be assessed during the baseline year (where already used) and at the end of the study period using WHO recommended assessment of durability. The association between integrity and protectiveness will be analysed.

3.7. Monitoring of interventions including sociological data

Cross-sectional surveys using questionnaire will assess user acceptability and perception of sustained IRS and LLINs. Questions asked will be to determine specifically what the villagers like and dislike about the interventions, whether they experienced any symptoms from the presence of insecticide, whether they have perceived any benefits from LLINs/IRS. IRS coverage is purportedly very high in the district at 96.7% which appears to indicate a high level of co-operation and user acceptance. Questionnaires will address specific issues of user fatigue after several spray rounds of IRS and perceived negative associations such as increase in other pests (an increase in fleas has been reported in some households).

Complementary to questionnaires, focus group discussions (FGD) with male and female household members from each arm of the trial will be carried out in collaboration with anthropologist/sociologist from NIMR Mwanza to assess initial responses to IRS, potential benefits and drawbacks compared to

current methods of protection (LLINs), and the reasons for non-compliance. A further round of focus group discussions during theintervention stage will consider perceptions of efficacy and persistence.

3.8. Data collection and analysis

Household questionnaires for the baseline and interventions cross sectional surveys will be programmed into PDAs and downloaded into Microsoft Access via Pendragon forms 5.1 software. This allows continual checking of the data without the need to wait for data entry. Entomological data will also be entered using PDAs. Clinical data will be recorded on paper in the field because these forms must be passed between the nurses doing the examination and the dispensing officer (clinician), who must approve each copy. The forms will then be double entered, validated and verified by data entry clerks in the PAMVERC office.

The full analytical plan will be developed later. Given the nature of the interventions it is not possible to conduct a blinded trial. Stratified randomisation of villages to the study groups will reduce the likelihood of chance imbalances between study arms but as only relatively small number of units can be randomized in such a cluster design, the 2 groups cannot be assumed similar for all factors. Data on parasite prevalence and anaemia will be collected for sampled children during baseline to adjust for any imbalances in malaria at clusters level. Only houses in the core sampling areas, situated atleast 1 km apart from the next cluster will be selected to reduce any spill-over effect (where an anti-vector intervention may also reduce or increase transmission in neighbouring villages).

Individual data will be collected on potential confounding factors to allow for adjustments in the final analysis. Potential confounders to be probed during cross sectional surveys include home treatment/prophylaxis of malaria, travel to other areas with different levels of transmission, and the use of other anti-mosquito measures which may prevent malaria transmission such as insecticidal sprays, coils, repellents, smoke, quality of LLINs by recording the number of holes and usage and quality of spraying (using pyrethroids detection kits). Household and personal data will be analysed with parasite prevalence data to identify and quantify individual, household and environmental risk factors.

3.9. Ethical considerations

Local ethical approval from KCMC has been granted (summarize comments from reviewers). Mandatory ethical clearance will be obtained from the Ethics Committees of KCMC, the National Institute of Medical Research and prior to initiating the study as indicated in the activity schedule. LSHTM ethics approval has been granted (number 5814).

Written informed consent will be obtained from the head of household or caregiver of each selected household. Community consent will be obtained from hamlet leaders.

3.10. Environmental Compliance standards

PMI will be responsible for the IRS programme including safe disposal of chemicals and deteriorated LLINs. We strive to conduct all research to international environmental standards. The project does

not involve the use of large quantities of disposable waste products. The clinical samples of blood, gloves, slides, and drug packaging will be disposed of according to standard protocols.

4. Work Plan

The trial will run for 2 years as per the detailed work plan and schedule. A comprehensive work plan and project schedule is outlined in this section (and is divided in 4 stages.

1. Stage 1: **The preparation** (Sept-Dec 2010) during which the project was established in Kagera region. A project manager (Dr Natacha Protopopoff) was recruited and based in Muleba. A project office was secured in Muleba as a headquarters for field activities, administration, reference laboratory and entomology. Project staff were recruited and trained. Coordination with district health authorities was achieved.

   The main technical activity during the first quarter was the mapping of the trial area consisting of more than 100 villages in which the study will be conducted. Each house in each hamlet was geo-referenced to facilitate the allocation of interventions and the sampling of households for cross sectional surveys and entomological surveillance. A detailed quarterly report covering this phase has been submitted.

2. Stage 2: **The pilot** (Jan to March 2011). Following the mapping conducted during the preparatory phases, 51 clusters comprising of 1 up to 3 villages were identified, and 100 houses randomly selected per cluster. A pilot cross sectional parasite survey to evaluate prevalence in the study area is ongoing and started in February. Based on the prevalence data the final selection of study clusters will be made. An IRS round just finished before the beginning of the pilot cross sectional survey.

3. Stage 3: **The baseline** (April-Sept 2011). During this stage it is anticipate that a distribution campaign of LLINs will occur beginning April with the objective to reach universal coverage. A second cross sectional prevalence survey (June-July), entomological monitoring, a feasibility evaluation of acquiring incidence data from health facility integration will be also conducted. The laboratory will be fully set up, SOPs for the different techniques written and staff trained.

4. Stage 4: **The intervention**, (Sept 2011-Aug 2012) anticipates IRS in Sept-Oct 2011 in half the clusters and distribution of LLIN to top up the universal campaign and reach a coverage and usage of minimum 80% in all the cluster. It will depend on whether RTI is sprayed in Sept 2011 or February 2012. Cross sectional prevalence surveys in Feb-March and again in June-July, with entomological and incidence monitoring from Jan-July will also be conducted.

Activity	Tool	Outcome measures	Target deadline
**Stage I – Preparation (September-December 2010)**			
Staff recruitment	Advertisements (already distributed)<br>Interviews (already scheduled)<br>Appointments made & contracts signed	Following staff in place: *Position (contracted to/based at)*<br>1. Trial manager (LSHTM/Muleba)<br>2. 8 Field Technicians (KCMC/Muleba)<br>3. Trial administrator (KCMC/Muleba)<br>4. Trial accountant (KCMC/Moshi)<br>5. Driver (KCMC/Muleba)<br>6. 2 security guards (KCMC/Muleba)<br>7. Data entry clerk (KCMC/Muleba)<br>8. 2 Laboratory technicians (KCMC/NIMR)<br>9. 4 Nurses and 2 clinical officers (temporary appointment/Muleba)	1. Achieved Q1.2<br>2. 7 employed Q1.1-1.2<br>3. Achieved Q1.1<br>4. Achieved Q1.1<br>5. Achieved Q1.1<br>6. Achieved Q1.1<br>7. To be identified in Q2<br>8. Identified in Q1.1<br>9. To be done in Q2 before survey<br>PhD student involved on the epidemiological part of the study Q2
Ordering equipment	Administrator LSHTM<br>Administrator KCMC (invoices, proforma etc)	*Purchase of major laboratory equipment:*<br>1. RT-PCR machine<br>2. 3x Laptop computers<br>3. 2x Microscopes<br>4. 20/20 Stand-alone reader<br>*Purchase of major field equipment:*<br>1. 5x GPS units<br>2. 5x Motorbikes<br>3. 5x PDA units (<br>4. 50 x light traps & batteries<br>5. RDTs and ACTs	1. Achieved Q2<br>2. 3 purchased Q2.1-1.2<br>3. Achieved Q1.2<br>4. Achieved Q1.1<br><br>1. Achieved Q1.1<br>2. Planned for Q2<br>3. Achieved Q1.2<br>4. Achieved Q1.2<br>5. To be done in Q2
Setting up offices	DMO Muleba to identify and assign an office	• Signed 1 year renewable contract agreement with DMO to rent one office within district administration site offices	Achieved Q1.2
	Meeting with landlady of private guesthouse (already identified)	• Signed 1 year renewable contract agreement to rent private guesthouse to equip for purpose of the project	Achieved Q1.2
	Assigning functions to the guesthouse rooms	• Office for project manager<br>• Office for project administrator<br>• Storeroom<br>• Mosquito sorting/identification room	Achieved Q1.2

		• Insectary<br>• 2 bedrooms for visiting project staff not based in Muleba<br>• Data management office	
	Buying and installing furniture	• Shelving for storeroom & offices<br>• Desks and filing cabinets for offices<br>• Fridge for mosquito sorting room<br>• Humidifier for insectary room<br>• Heater and/or A/C unit for insectary room	Achieved Q1.2, with the climate condition in Muleba Humidifier and heater not necessary.
Field technician training	2-day GPS mapping course	• Proficiency in marking, saving, editing and deleting waypoints, using the track logger, finding waypoints & using routes, using pre-determined project waypoint and track naming system, abbreviations and symbols	Achieved in Q1.2 with knowledge used extensively during the mapping of clusters
	1 week intensive motorbike proficiency course	• Licenses for all field technicians<br>• Certification from Muleba driving school	Plan for Q2 before field work
	1-day slide staining course	• Slide preparation training for the 2 laboratory technicians at KCMC	On going in Q1.2 and will continue during Q2
Information dissemination and community sensitisation	Group meeting with village leaders, hamlet leaders and household heads	• Community understanding of stage one and stage two activities; verbal agreement to participate and support project activities	Sensitization for mapping done in Q1.2 and additional sensitization are planned before the beginning of the pilot in Q2
	Reporting to HRCD	• Quarterly technical report including Performance Monitoring Report	Table part of the Q1.1-Q1.2
Stakeholder coordination	Meeting(s) with HQ representatives PMI/RTI/NMCP/WHO	• Established schedule of face-to-face and/or teleconference appointments to update project partner institutes<br>• Identify representatives from each institute to attend, as well as locations and meeting structure (quorum etc.)	Individual contact has been done during the past quarter with every partner. Meeting will be held during Q2
Coordination Committee	Discussions with Kagera-based representatives	• Establishing Coordination Committee	To be done in Q2
Study area identification	GPS	• Geo-location data for all village centres within 45min drive of Muleba town<br>• Map main roads within study area	Identification done in Q1.2

		• Identify divisions and wards within study area	
Health Facility (HF) Involvement	Assessment of potential involvement as vehicle for incidence proxy	• Concept agreement with stakeholders<br>• Mapping of HF locations within study area<br>• Determining catchment area for each HF<br>• Establish inclusion criteria<br>• Tabulation of health records and level of usage by catchment population	Mapping of HF within the study areas done in Q1.2 and collection of malaria cases in some of them. Full assessment will be done when regular supply of RDTs can be assured.
**Stage II – Pilot (January-April 2011)**			
IRS in February 2011 in all clusters			
Mapping houses and village boundaries	GPS units and ExpertGPS 3.8 software program (TopoGrafix)	• Geo-location data for all houses in a minimum of 50 villages<br>• Demarcation (boundary tracks) of the same 50 villages<br>• Computer-generated map of all houses and tracks	Started in Q1.2 and completed in Q2
Sensitization	<u>Meeting with ward, village and hamlet leader</u>	• The hamlet leader are informed about the purpose of the study and fully involved during the cross sectional survey and the entomological monitoring	Feb 2011
Cross-sectional pilot survey *(100 children 0.5 to 14 years from ~100 households in each of 51 clusters)*	Detection of *Pf* infection by RDTs, bloodslides, PCR	• 5000 children surveyed<br>• 5000 slides stained and double/single-read<br>• Data entered from RDTs<br>• *Pf* % prevalence by age group and sampling technique<br>• Test the PCR methods of detection of *Pf* from bloodspots	End Feb 2011<br>End Apr 2011<br>“<br>“
	Simplified household questionnaire including question on bednet usage and IRS coverage	• % of children who slept under a bednet on the previous night<br>• % of parents/guardians who recall IRS was conducted in their house in 2011	End April 2011<br>“<br>“
Sociology study	Focus Group Discussion	• Perception of LLIN and IRS	April 2011

		• Determinant of non adherence or none used to IRS or/and LLINs	
Selection finalization of clusters	Selection criteria:<br>a) Distance from project centre in Muleba town<br>b) Distance between clustersd) Parasite prevalence<br>e) Sample size requirements	• Identification of sufficient number of clusters to meet sample size requirement (current estimate = 30)	Mid May 2011
Setting up the Laboratory in Moshi	Entomology<br>• Real time PCR technique<br>• ELISA for sporozoite and blood meal<br>Clinical<br>• Serology techniques<br>• Sub microscopy	• Equipment is received<br>• Trining of laboratory staffs on the different techniques<br>• SOPs are ready	March-April 2011
Mosquito trapping	One night collection in 10 houses in each of the 51 with light traps (5 houses) and spray sheet collection (5 houses)	• Abundance and relative proportion of *Anopheles gambiae* and *An. funestus* sibling species<br>• Selection of clusters to be included in the resistance study<br>• Selection of the best trapping methods for the area	Mid April 2011<br><br>End May 2011
**Stage III – Baseline year (June-September 2011)**			
Cross-sectional survey *(100 children from ~100 households in each of ~30 clusters)*	Detailed household questionnaires<br>including detailed question on IRS coverage, and LLIN usage	• Completion of questionnaire survey in ~100 houses within each of 30 clusters<br>• Household census & risk factors data<br>- Socioeconomic data<br>- Bednet usage; IRS and other insecticide use, perception of different intervention methods<br>• % of children who slept under an ITN/LLIN on the previous night<br>• % of households with evidence of IRS in February2011, e.g. adult recall, IRS	June-July 2011<br><br>September 2011

		- House structural data	
	Detection of *Pf* infection by RDTs, bloodslides, PCR	• 3000 children surveyed<br>• XXXX adult surveyed<br>• 3000 RDTs taken and read<br>• 3000 slides stained and double read<br>• PCR detection of *Pf* from bloodspots<br>• *Pf* % prevalence by age group and diagnostic technique	June-July 2011<br>“<br>“<br>September 2011<br>“<br>“
	Sero-prevalence (bloodspot ELISA)	• Impact of IRS on sero-conversion rates by age group and spray history	September 2011
Entomology	Longitudinal monitoring (Light trapping and space spray)	• Abundance, infectivity and relative proportion of *Anopheles gambiae* and *An. funestus* sibling species within and between clusters<br>• Impact of IRS and LLIN on EIR	May-Dec 2011
	Resistance	• Susceptibility levels by species<br>• Detection of resistance by genotypes e.g. *kdr*	May-Dec 2011
	Assessment of IRS longevity	• Pyrethroid detection kit<br>• Household acceptability	May-June 2011 (during cross sectional survey)
Coordination with control programme	National and local coordination meetings PMI/RTI/NMCP/WHO/ MoH	• Information sharing<br>• Resource management<br>• Coordination of intervention and monitoring activities	National – Quarterly<br>Local - monthly
Incidence measures	Incorporation of health facilities into monitoring programme	• Evaluation of feasibility and cost<br>• Orientation of HF staff<br>• Strengthening of diagnostics (e.g. RDT availability and proper usage, microscopy) and treatment (e.g. ACT availability)<br>• Training in record management and reporting<br>• Analysis of records as incidence proxy	August 2011<br>Will depend of the feasibility
**Stage IV – Intervention year (September 2011-August 2012)**			
Timeline anticipates IRS in Sept-Oct 2011 in half of clusters (subject to change) and LLIN distribution to top up coverage in all clusters			

Sensitization	<u>Meeting with ward, village and hamlet leader</u>	• The hamlet leader are informed about the purpose of the study<br>• The hamlet leader are explained which one will get witch intervention	
LLIN distribution top up (MEDA/NMCP/LSHTM,KCMC,NIMR)		• Determination of net requirement by cluster<br>• Planning distribution activities<br>• Training for implementation<br>• Distribution<br>• Monitoring of coverage	August 2011<br>Sept-Oct 2011
Cross-sectional post intervention survey *A* *(100 children from ~100 households in each of ~30 clusters)*	Questionnaires	• Completion of questionnaire survey in ~100houses within each of 30 clusters<br>• Household census & risk factors data<br>- Socioeconomic data<br>- Bednet usage; IRS and other insecticide use<br>• % of children who slept under an ITN/LLIN on the previous night<br>• % of households with evidence of IRS in September 2011, e.g. adult recall, IRS<br>- House structural data	Jan-Feb 2012<br><br><br><br>Feb-March 2012
	Detection of *Pf* infection by RDTs, bloodslides, PCR	• 3000 children surveyed<br>• 3000 RDTs taken and read<br>• 3000 slides stained and double read<br>• PCR detection of *Pf* from bloodspots<br>• *Pf* % prevalence by age group and diagnostic technique	Feb-March 2012<br>“<br>End April 2012<br>“<br>“
	Sero-prevalence (bloodspot ELISA)	• Impact of IRS on sero-conversion rates by age group and spray history	May 2012
Cross-sectional post intervention survey *B* *(100 children from ~100households in each of ~30 clusters)*	Questionnaires	• Completion of questionnaire survey in ~7100houses within each of 30 clusters<br>• Household census & risk factors data<br>- Socioeconomic data<br>- Bednet usage; IRS and other insecticide use<br>• % of children who slept under an ITN/LLIN on the previous night<br>• % of households with evidence of IRS in	April-May 2012

		September 2011, e.g. adult recall, IRS<br>- House structural data	
	Detection of *Pf* infection by RDTs, bloodslides, PCR	• 3000 children surveyed<br>• 3000 RDTs taken and read<br>• 3000 slides stained and double read<br>• PCR detection of *Pf* from bloodspots<br>• *Pf* % prevalence by age group and diagnostic technique	May-June 2012<br>"<br>July 2012<br>"<br>"
	Sero-prevalence (bloodspot ELISA)	• Impact of IRS on sero-conversion rates by age group and spray history	July 2012
Sociological study	Focus Group Discussion	• Perception of LLIN and IRS<br>• Reason of non adherence or used to IRS or/and LLINs	April 2012
Entomology	Longitudinal monitoring (Light trapping and space spray)	• Abundance, infectivity and relative proportion of *Anopheles gambiae* and *An. funestus* sibling species within and between clusters<br>• Impact of IRS on EIR	Jan-July 2012
	Resistance	• Susceptibility levels by species<br>• Detection of resistance by genotypes e.g. *kdr*	Jan-July 2012
	Assessment of IRS longevity	• Pyrethroid detection kit<br>• Household acceptability	Post-spray bimonthly
Coordination with control programme	National and local coordination meetings PMI/RTI/NMCP/WHO/ MoH	• Information sharing<br>• Resource management<br>• Coordination of intervention and monitoring activities	National – Quarterly<br>Local - monthly
Incidence measures		• Continuation of HF activities as per baseline	Jan-July 2012
Trial Impact	Data analysis	• Epidemiological impact<br>-cross sectional prevalence<br>-incidence<br>• Entomological outcomes<br>-EIR<br>-Resistance	Jan-September 2012

		• Community acceptability	
	Dissemination of findings	• Community meetings<br>• Publications<br>• Annual reports	October 2012
