# Supplementary material for: Enhanced Protection against Malaria by Indoor Residual Spraying in Addition to Insecticide Treated Nets: Is It Dependent on Transmission Intensity or Net Usage?
Source: PLoS One. 2015 Mar 26;10(3):e0115661. doi: 10.1371/journal.pone.0115661 (PMC4374910; doi:10.1371/journal.pone.0115661)
Supplement: S2 Study Protocol — (PDF) [file pone.0115661.s003.pdf]

Amendments to Protocol of the PAMVERC malaria prevention trial: Muleba

1. **Malaria Incidence as outcome indicator:**

   It was investigated in 2010 whether it was possible to use passive case detection through the primary health care system as an indicator of malaria incidence. Evidence of frequent stock-outs of RDTs in health facilities in this area at that time guided the decision not to use passive case detection data from health facilities since reporting of cases was not always based on definitive diagnosis.

Agreed by Steering committee on 30/06/2011

2. **Study arms of trial: ITNs plus IRS versus ITNs alone:**

   The whole of Tanzania, including the study area, was supplied with LLINs in a universal coverage campaign between 2010 and 2011. An IRS only arm as the reference arm of the study, as originally envisaged in the protocol, is no longer possible since ITNs (LLINs) at high coverage are now the standard of care in Tanzania. The study arms would therefore be: (1) ITNs only (reference arm), and (2) combined use of ITNs and IRS.

Agreed by Steering committee on 30/06/2011

3. **Insecticide policy:**

   The insecticide to be used for IRS to be changed from one round of lambda-cyhalothrin annually to two rounds of bendiocarb annually, from 2012.

   Reason for change: Documentation of high levels of phenotypic pyrethroid resistance in An. Gambiae in July 2011 (determination of the resistance mechanisms kdr and oxidases was established later, in March 2012). Short residual of bendiocarb on walls, requires two rounds of IRS to cover both seasons. Round 1 to be sprayed between December 2011 and January 2012. Round 2 to be sprayed between April and May 2012 (round 2)

Agreed by Steering committee on 31/09/2011

4. **Number of surveys:**

   One additional cross-sectional household survey to be conducted in November 2012. Reason for change: The second survey was originally planned at six months or longer after IRS. With the second round of IRS the interval between the last spray round and the survey would be much shorter (2 months). The third survey to be conducted approximately 6 months after the last round of IRS and 10 months after the first round.

Agreed by Steering committee on 31/03/2012

5. **Analysis as a superiority study:**

   The trial with revised study arms should answer the following specific question: Is the use of IRS and ITNs superior to using only ITNs?

Agreed by Steering committee on 31/03/2012
